# Temporal dynamics of microbial transcription in wetted hyperarid desert soils

**DOI:** 10.1093/femsec/fiae009

**Published:** 2024-01-31

**Authors:** Carlos León-Sobrino, Jean-Baptiste Ramond, Clément Coclet, Ritha-Meriam Kapitango, Gillian Maggs-Kölling, Don A Cowan

**Affiliations:** Centre for Microbial Ecology and Genomics, Department of Biochemistry, Genetics and Microbiology, University of Pretoria, 0002 Pretoria, South Africa; The Novo Nordisk Foundation Center for Biosustainability, Technical University of Denmark, 2800 Kgs Lyngby, Denmark; Centre for Microbial Ecology and Genomics, Department of Biochemistry, Genetics and Microbiology, University of Pretoria, 0002 Pretoria, South Africa; Extreme Ecosystem Microbiomics and Ecogenomics (E²ME) Lab., Facultad de Ciencias Biológicas, Pontificia Universidad Católica de Chile, Santiago, Chile; Centre for Microbial Ecology and Genomics, Department of Biochemistry, Genetics and Microbiology, University of Pretoria, 0002 Pretoria, South Africa; Gobabeb – Namib Research Institute, Walvis Bay, Namibia; Gobabeb – Namib Research Institute, Walvis Bay, Namibia; Centre for Microbial Ecology and Genomics, Department of Biochemistry, Genetics and Microbiology, University of Pretoria, 0002 Pretoria, South Africa

## Abstract

Rainfall is rare in hyperarid deserts but, when it occurs, it triggers large biological responses essential for the long-term maintenance of the ecosystem. In drylands, microbes play major roles in nutrient cycling, but their responses to short-lived opportunity windows are poorly understood. Due to its ephemeral nature, mRNA is ideally suited to study microbiome dynamics upon abrupt changes in the environment. We analyzed microbial community transcriptomes after simulated rainfall in a Namib Desert soil over 7 days. Using total mRNA from dry and watered plots we infer short-term functional responses in the microbiome. A rapid two-phase cycle of activation and return to basal state was completed in a short period. Motility systems activated immediately, whereas competition–toxicity increased in parallel to predator taxa and the drying of soils. Carbon fixation systems were downregulated, and reactivated upon return to a near-dry state. The chaperone HSP20 was markedly regulated by watering across all major bacteria, suggesting a particularly important role in adaptation to desiccated ecosystems. We show that transcriptomes provide consistent and high resolution information on microbiome processes in a low-biomass environment, revealing shared patterns across taxa. We propose a structured dispersal–predation dynamic as a central driver of desert microbial responses to rainfall.

## Teaser

Structured and rapid microbiome response to watering in a Namib Desert soil using metatranscriptomics.

## Importance

Desert microbiology is a field attracting an increasing research interest in the light of ongoing global aridification and next-generation agricultural research. In these ecosystems, the effect of rare precipitation events is one of major importance. In this study, we analyze the soil microbiome response of the Namib Desert to simulated rainfall. Our approach targeted mRNA as a dynamic indicator of active functions, whereas most microbiome surveys focus on community structure and functional potential. Our results demonstrate the potential of metatranscriptomics to robustly capture short-term microbial processes in a natural environment, even from complex microbiomes and low-biomass samples. We report a sequential, structured, and dynamic functional response completed in 7 days, which we condensed into a dispersal–predation cycle model.

## Introduction

Arid lands cover approximately one-third of the terrestrial surface (Laity 2009) and are currently expanding through desertification processes (Huang et al. 2016). (Hyper)Aridity, simultaneously caused by very low precipitation and high potential evapotranspiration rates, severely limits biomass production, and leads to soil nutrient depletion (Delgado-Baquerizo et al. 2013, Maestre et al. 2015), resulting in habitat fragmentation where productivity is concentrated in sheltered ‘islands’ (Schlesinger et al. 1995, Pointing and Belnap 2012). Microbial biomass is likewise constrained, sometimes forming macroscopic structures such as biological soil crusts (BSCs) and hypoliths. Open soils outside of these privileged microenvironments are more extreme and subjected to an intense desiccation stress which limits biological activity (Lebre et al. 2017). Despite this, microorganisms in these unsheltered areas are responsive to rare pulses of water (Garcia-Pichel and Pringault 2001, Austin et al. 2004, Collins et al. 2014, Armstrong et al. 2016, Štovíček et al. 2017) and may be important for long-term soil fertility and postrainfall grass germination (Delgado-Baquerizo et al. 2016).

In this environment, the expected reduced activity—or dormancy—of microbes during the long dry periods and the preservation of legacy DNA (Austin et al. 2004, Lennon et al. 2018), can be confounding factors for the study of microbiomes. Transcriptomics thus represents a powerful tool by providing a direct insight into microbial activity due to the ephemeral nature of mRNA, going beyond community structure or genetic potential analyses (Moran et al. 2013, Rajeev et al. 2013, Štovíček et al. 2017, Steven et al. 2018, León-Sobrino et al. 2019).

Given the importance of rare and stochastic water pulses in driving ecosystem functions in exposed desert soils (e.g. Pointing and Belnap 2012, Armstrong et al. 2016), we aimed to identify the temporal dynamics of microbial community responses to wetting in these water-deficient desert soils. We hypothesize that the activation of key cellular functions is a factor in the adaptation of desert soil microbiomes to these extreme conditions. A representative gravel plain soil from the central Namib Desert was subject to an artificial rainfall pulse of 30 l/m^2^, sufficient to stimulate plant germination (Seely and Pallett 2008). The short-term responses of the soil microbiome were monitored by analyzing gene function through mRNA sequencing. As a result of this analysis, we propose a structured water response model with differentiated phases and trophic interactions, which may serve as a basis for improved, function-oriented analyses of microbiomes in arid ecosystems.

## Materials and methods

Subsurface soils were collected from two adjacent 3.5 m × 3.5 m plots (∼10 m apart) in the central Namib Desert gravel plains (23°33′18″S, 15°3′20″E, or −23.505°, 15.056°). The control plot remained dry, while the experimental plot was manually watered with 30 l/m^2^, the approximate average annual rain received in this region (25 mm) (Eckardt et al. 2013), at *T*_0_ (26 April 2017 10 a.m. WAT/UTC+1), using a synthetic ‘Namib Desert rain’ solution, prepared from ultrapure DNA-free water supplemented with a defined salt mixture (Frossard et al. 2015). Plots were subdivided into 0.5 m × 0.5 m quadrats and randomly sampled, in triplicate, at specified times after water addition (Figure S1, Supporting Information). A total of 20 g samples of surface (0–5 cm) soil were collected at 10 min; 1, 3, and 7 h; and 1, 3, and 7 days after simulated rainfall, preserved immediately in RNAlater solution (Sigma-Aldrich, St. Louis MO, USA) and subsequently frozen at −20°C prior to RNA extraction. Additional 200 g soil samples were collected from the same locations into WhirlPak™ bags (Nasco, Fort Atkinson WI, USA) and frozen for subsequent soil chemistry analysis.

The water content of soil samples (sieved to < 2 mm particles) was measured gravimetrically for 3 days after watering. Soil silt, sand, and clay compositions were measured by the hydrometer method (Gee and Bauder 1986). Particle size distributions were determined by sieve separation (> 1000 µm, > 500, > 250, > 100, > 53, and < 53 µm). pH, electrical conductivity, Na, Cl, K, Ca, Mg, NO_3_, NH_4_, and P composition were analysed at Bemlab (Pty) Ltd. (Strand, Western Cape, South Africa) using standard protocols. Soil organic carbon percentage was measured using the Walkley–Black test (Walkley 1935).

RNA was extracted following protocols described previously (León-Sobrino et al. 2019) from triplicate soil samples. Briefly, RNA was purified using TRIzol and treated with DNase I, followed by precipitation in 2 M LiCl to ensure complete elimination of genomic material. In order to mitigate the effect of soil chemistry heterogeneity, the two biological replicates most similar to the average composition of all sampled soils were selected for RNA extraction. Stranded, rRNA-depleted libraries were prepared with the ScriptSeq Complete Gold Kit (Epidemiology) (Illumina, San Diego, USA) and 150 bp paired-end sequences were read on a HiSeq4000 platform (Illumina).

Sequencing outputs were processed using the BBtools suite v. 38.26 (Bushnell et al. 2017) (https://sourceforge.net/projects/bbmap/). Read ends below a quality Phred value of 20 were trimmed using *BBDuk*; rRNA and human RNA sequences were identified and removed using SILVA v. 111 (July 2012) (Quast et al. 2012) and 5SRNA (Szymanski et al. 2016) databases and a curated human genome reference assembly hG19 (https://drive.google.com/file/d/0B3llHR93L14wd0pSSnFULUlhcUk) (bbmap 2018) following recommended protocols. Optical duplicates generated by the patterned sequencing flowcell were removed using the *Clumpify* function from the BBtools suite, setting the distance cut-off to 2500 pixels. Transcript assembly was performed using transAbyss v.2.0.1 (Robertson et al. 2010) for each library. Individual assemblies were merged with the same software (*transabyss-merge* function) to generate a reference metatranscriptome.

Contigs were annotated at the Integrated Microbial Genomes and Microbiomes (IMG/M) server (Huntemann et al. 2015) (https://img.jgi.doe.gov/). Predicted protein products from genes were also analysed against the Conserved Domain Database (CDD) using Delta Blast (e-value threshold 10^−4^) (Boratyn et al. 2012, Marchler-Bauer et al. 2015). Contig taxonomy was determined by a consensus among all individually classified genes, requiring a *quorum* of > 50% at each taxonomic level.

Reads were aligned to the reference assembly using BBmap (Bushnell et al. 2017) and reads for annotated regions in each library were counted using FeatureCounts v. 1.6.3 (Liao et al. 2014). The assembled counts matrix was aggregated along functional and/or taxonomic categories as required for each analysis.

Differential transcription along the time series was analysed with the DESeq2 v. 1.14 package in R (Love et al. 2014), comparing data from the control (dry) soil samples with those from the experimental (wetted) samples from the same point in the time-series. Transcripts that significantly diverged in abundance from the control at any given time were considered upregulated in response to watering (adjusted *P*-value ≤ .05 for the likelihood ratio test). Transcripts per million (TPM) of ribosomal protein genes as a fraction of the total for their respective taxonomic group (Rp: T) were employed to estimate absolute activity of each group along the time course, following the premise that ribosome densities in a cell relate to metabolic activity and growth rates (Bremer and Dennis 1996, Bosdriesz et al. 2015).

Viral contigs were identified from assembled transcriptomes using VirSorter v. 1.0.3 (Roux et al. 2015) and the virome database on the iVirus platform hosted by CyVerse (Bolduc et al. 2017). Only contigs >1 kb, and classified as categories 1, 2, 4, and 5 were considered (phages and prophages identified with the ‘pretty sure’ and ‘quite sure’ qualification). To calculate the relative abundances of the different viral contigs in each transcriptome, quality filtered metatranscriptomic reads were mapped back to the viral contigs with Bowtie2 v. 2.2.6, using the default parameters (Langmead and Salzberg 2012). The output SAM files were converted into BAM files, sorted and indexed, using SAMtools (Li et al. 2009).

ORFs were predicted within putative viral contigs using Prodigal (Hyatt et al. 2010). TPM were employed to normalize the final ‘viral OTU’ (vOTU) values for each sample. Predicted protein sequences were clustered with proteins from viruses in the NCBI ViralRefSeq-prokaryotes-v85 based on all- versus-all BLASTp search with an E value of 1 × 10^−3^, and clusters were defined with the Markov clustering algorithm and processed using vConTACT2 (Bin Jang et al. 2019). The stringency of the similarity score was evaluated through 1000 randomizations by permuting protein clusters or singletons (proteins without significant shared similarity to other protein sequences) within pairs of sequences having a significance score ≤ 1 (negative control). Subsequently, pairs of sequences with a similarity score > 1 were clustered into VCs with the Markov clustering algorithm using an inflation value of 2. The resulting gene-sharing network from vConTACT2 classification was visualized with Cytoscape software v. 3.7.0 (Smoot et al. 2011). Reference sequences from RefSeq database that coclustered with the putative viral sequences were used to predict viral taxonomy.

## Results

### Site characteristics and taxonomic analysis

**Figure 1.**
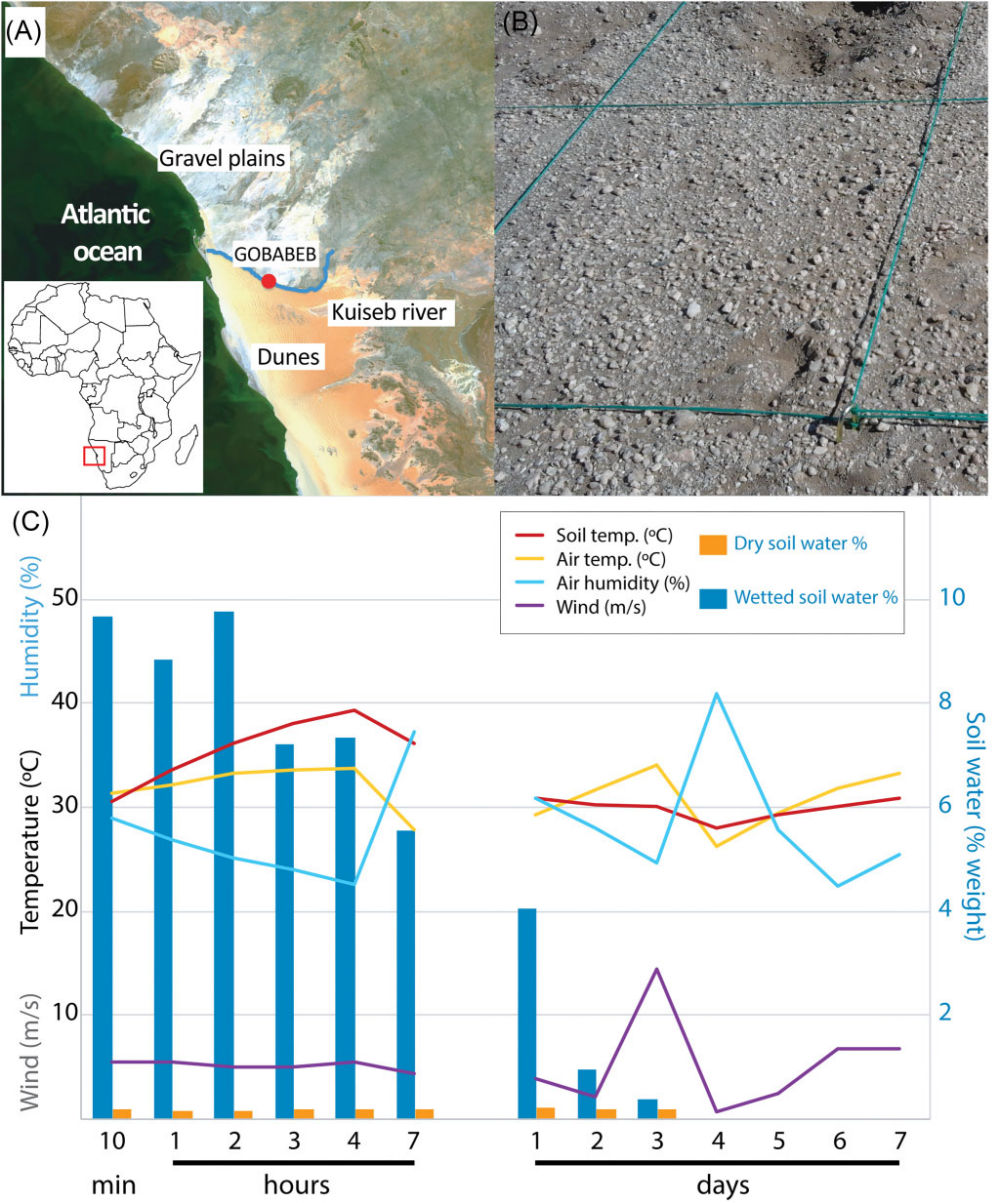
Sample site and environmental conditions of the sampled soils. (A) Map of the Namib Desert gravel plain location. Modified from European Space Agency, ESA/Envisat CC BY-SA 3.0 IGO. (B) View of a representative portion of the gravel plain sampling site. (C) Environmental conditions over the experimental period: Air temperature, humidity, and wind were recorded by the nearby Gobabeb meteorological station [Southern African Science Service Centre for Climate Change and Adaptive Land Management (SASSCAL), station 8893].

The sample site (Fig. 1A and B) is a characteristic Namib Desert calcrete gravel plain (Gombeer et al. 2015) with high sand composition (92 ± 1.6%) and very low organic carbon content (0.04%) (Table S1, Supporting Information). Soil composition was relatively homogeneous in all sampled sectors and between the sample and control sites (Table S1, Supporting Information). Gravimetric water content measurements showed that more than half of the water content in the surface soils (0–5 cm) was lost 24 h after the simulated 30 mm rainfall. After 3 days, the water content was similar to that of the dry control (Fig. 1C).

Transcript read assembly yielded a consensus metatranscriptome of 208.95 Mb in which 378 802 coding regions were annotated, including 372 044 predicted protein-coding genes. On average, for the 24 sequenced libraries, 61.7% of reads could be aligned back to contigs. Functions were predicted for 29.9% of the protein-coding genes using the KEGG database (Kanehisa et al. 2016), and 51.6% using the CDD. 56.8% of contigs were taxonomically classified at phylum level, and 56.3% down to family level.

**Figure 2.**
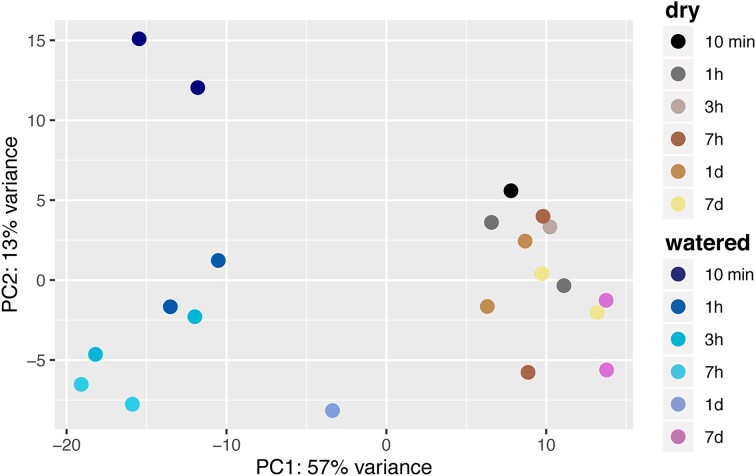
Principal components analysis of transcriptomes according to KEGG Ortholog functional annotations across soil samples.

A functional and taxonomic analysis of the transcriptional profiles revealed large differences in gene expression levels between treatment and control soil transcriptomes within 10 min after watering (Fig. 2; Figure S2, Supporting Information). Communities from dry soils were characterized by stable (i.e. largely unchanged) transcription profiles over the 7-day experimental period. In contrast, microbial communities in the watered soils underwent an abrupt change in gene expression (Fig. 2) that progressively returned to the basal state within the 7 days of the experiment.

In both watered and dry soils, Actinomycetota and Pseudomonadota were the most transcribed phyla (40% and 16% in average, respectively, see Table S2, Supporting Information). Strikingly, transcripts from Nitrososphaeria, a class of ammonia oxidizing Thaumarchaeota, were the third most abundant (average 14% of classified TPM in all samples). Protist transcripts (Oligohymenophorea class and Dictyostellales), which were rare in dry soil samples (< 0.5%), increased to > 4% within 7 h of soil watering. Conversely, transcripts of fungal origin were more common in dry soils (1% of classified TPM) than in watered soils.

**Figure 3.**
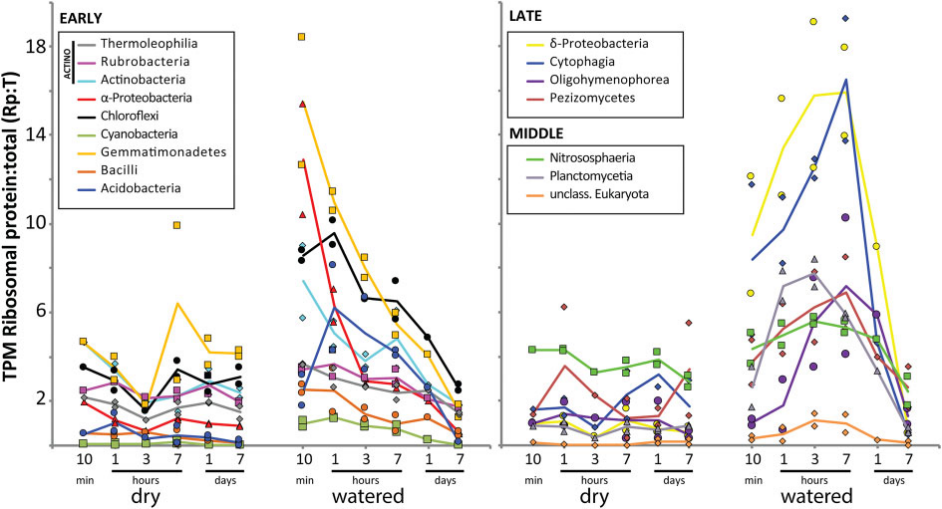
Ribosomal protein gene transcripts as a fraction of the total (Rp: T) among the most transcriptionally active microbial classes in the Namib soil community (> 1% TPM average in dry or watered samples). Left panel shows *early* response classes whose Rp: T-ratios peak within the first hour after watering. Right panel includes classes with Rp: T maxima 3 and 7 h after watering (*middle* and *late* response taxa, respectively).

The production of ribosomes in each taxonomic group was estimated by calculating the ratio of ribosomal protein transcripts with respect to the total (Rp: T) (Bremer and Dennis 1996, Bosdriesz et al. 2015), and used as an indicator of overall metabolic activity. This ratio was stable for each taxon in the dry control samples, typically < 4% TPM (Fig. 3). In contrast, after water addition, Rp: T increased in all the major taxa, peaking at characteristic times over the course of the experiment and returning to basal values by the end of the 7 day period. Actinomycetia, Alpha-proteobacteria, and Chloroflexia were representatives of ‘early-activation’ groups, with Rp: T-values peaking within the first hour. Gemmatimonadetes experienced the largest relative increase in Rp: T, despite being a minor component of the soil microbiome (< 1% TPM). A delayed rise in activity, reaching the highest values 7 h after the watering, was evident for Delta-proteobacteria, protists and Bacteroidota, especially those belonging to the Cytophagia class, which constituted the principal ‘late-activation’ group. The Pezizomycetes, the most transcribed fungal class, also responded late to water addition. Intermediate patterns, with maximal Rp: T-values at 3 h after water addition, were observed for taxa such as Planctomycetia. Thaumarchaeal Rp: T-ratios showed only modest changes after watering and a relatively even Rp: T throughout the experiment.

### Temporal changes in core cellular functions

**Figure 4.**
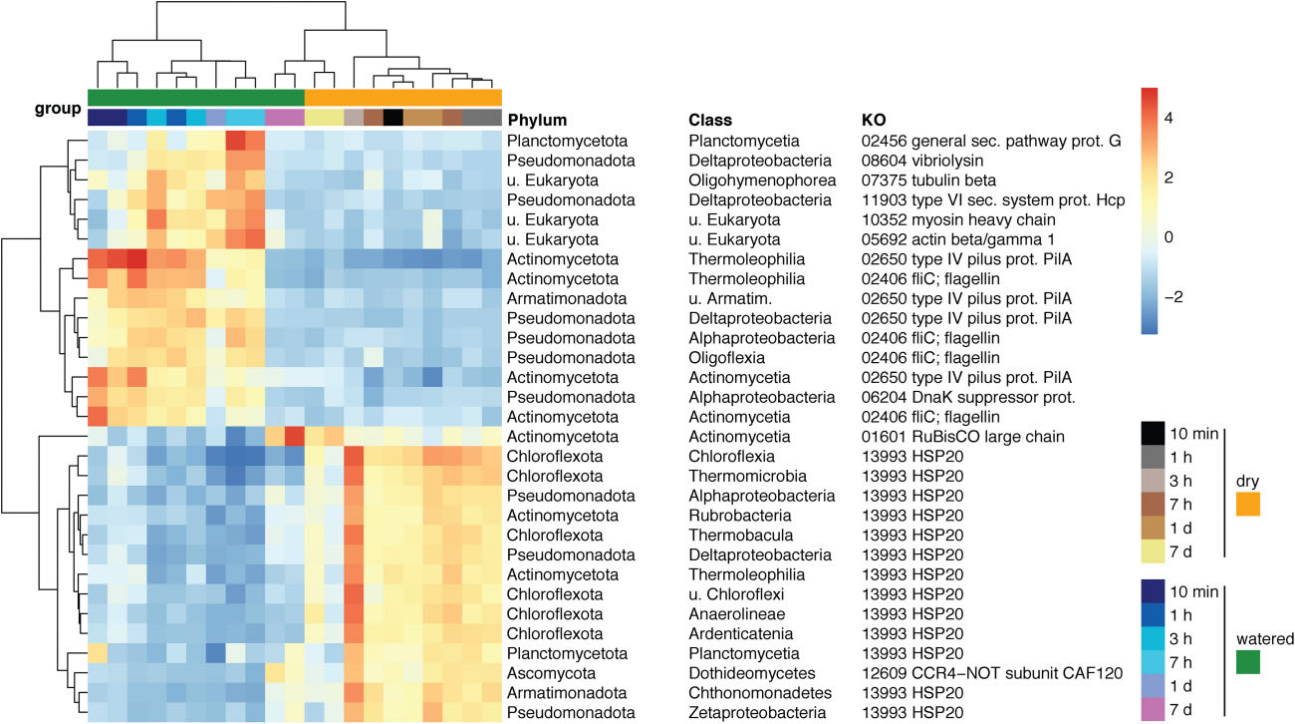
Temporal transcription patterns of the 30 most variable genes among those with significantly differential transcription along the time series. Transcript data was aggregated along taxonomic (class) as well as functional (KEGG Orthologs) groups for the analysis. Values were normalized using the Variance Stabilizing Transformation (*DESeq* R package).

A general reduction of stress resistance gene transcripts (e.g. *groEL, dnaK, clp*, and *tre*) was observed immediately after watering. Trehalose synthesis genes (*tre*), which drive solute accumulation under conditions of water stress (Lebre et al. 2017), declined in relative abundance in watered soils. Transcripts for chaperones *groEL* and *dnaK*, and the *clp* protease involved in protein misfolding control were also reduced. The most conspicuous change in core stress resistance systems was, however, that of the heat-shock protein HSP20 genes (KO13993, CD223149, or CD278440). Genes for this ATP-independent chaperone experienced the largest and most consistent transcript reduction across all significant (average ≥ 1% of transcripts) bacterial taxa after watering (Fig. 4).

Motility related transcripts were rapidly affected by soil wetting. Flagellar gene expression was upregulated within the first hour in the dominant Actinomycetota and Alpha-proteobacteria taxa (Fig. 4) and in other taxa such as Planctomycetota and Bacillota. Expression of type IV pilus genes, involved in gliding motility, was also rapidly upregulated after water addition, most notably in Actinomycetota. Simultaneously, hallmark chemotaxis genes *mcp* and *motB* were upregulated during the initial period in Actinomycetota, Proteobacteria, Bacteroidota, and Planctomycetota. A significant upregulation of Eukaryotic adhesion and cytoskeletal components, myosin, actin, and tubulin, was evident 3–7 h after soil wetting (Fig. 4), but was limited to nonfungal taxa, particularly in Oligohymenophorea (ciliate) and Dictyosteliales (slime mould).

Another highly significant increase in transcriptional activity observed 3–7 h after water addition involved interbacterial competition and predation genes. These included type VI secretion systems (T6SS) and vibriolysin genes from the order Myxococcales (Delta-proteobacteria), and T6SS and serralysin genes from Alpha-proteobacteria. Heightened T6SS transcription was also observed in several other bacterial taxa, such as Planctomycetota, Gemmatimonadota, and Gamma-proteobacteria.

### Biogeochemical cycles

#### Carbon utilization

Microbial photosynthetic processes are limited in hyperarid soils, but are strongly activated after wetting (Warren-Rhodes et al. 2006, Tracy et al. 2010, Gwizdala et al. 2021). However, surprisingly, transcript data did not show a significant relative increase in overall cyanobacterial transcripts across the 7-day duration of the experiment. Cyanobacterial Rp: T-ratios remained low throughout (Fig. 3; Table S2, Supporting Information), suggesting that cyanobacterial metabolism was largely unaffected by water addition in these soils.

Core carbon-fixation genes, including RuBisCO and carboxylase genes from chemoautotrophic pathways, consistently showed reduced relative transcription in wetted soils. Thaumarchaeal carbon fixation was seemingly also affected by watering, reflected in the transient inhibition of 4-hydroxybutyryl-CoA dehydratase from the 3-hydroxypropionate/4-hydroxybutyrate cycle following inundation. Overall, we observed an inhibition of both photosynthetic and nonphotosynthetic carbon fixation mechanisms immediately after soil saturation (Figure S3A, Supporting Information). Conversely, transcription of carbon fixation genes recovered by the end of the 7-day experimental period, after soil water content had returned to basal levels (Fig. 4; Figure S3A, Supporting Information).

Dramatic increases in CO_2_ emissions from newly wetted desert soils, attributed to degradation of dissolved organic matter, have been widely reported (Austin et al. 2004). Soil chemistry analyses, however, showed no significant reduction in soil Total Organic Carbon pools (Table S1, Supporting Information), possibly due to the very low organic carbon levels present in these soils. In our transcript data, indicators of biomass degradation, including carbohydrate and peptide transport systems (e.g. *rbsB, xylF*, and *livK, dppA*) significantly increased immediately after water addition. The Bacteroidota phylum, dominated by the Cytophagales, showed dramatic upregulation of subtilisin protease transcripts and components of the protein and carbohydrate import machinery (*ragA*/*susC* CD274948; *susD* CD185760).

#### Nitrogen and phosphate utilization

**Figure 5.**
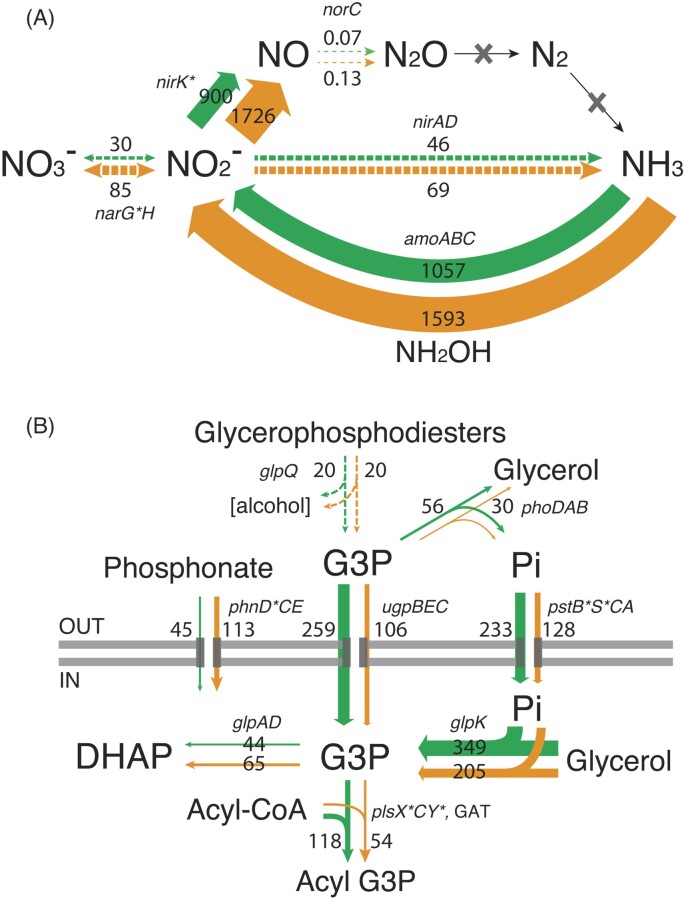
(A) Nitrogen cycling and (B) phosphate assimilation system transcription in the subsurface soil microbial community. Average TPM measurements are provided for the dry plot samples (orange arrows) and wetted samples up to 7 h postwatering (green arrows). Asterisks indicate significant differentially transcribed KOs at community level at any point along the time series. Panel (B) figure modified from León-Sobrino et al. (2019).

Transcript data suggested that Thaumarchaea were the main drivers of nitrogen cycling in the soil. Ammonia monooxygenase and NO-forming nitrite reductase (*amo* and *nirK*) transcripts, originating largely from Thaumarchaea, were among the most abundant overall. These declined in relative abundance after soil watering (Fig. 5A; Figure S3B, Supporting Information). All other transcripts implicated in ammonification, such as nitrate and nitrite reductases *narGH* and *nirAD* (mainly transcribed by Nitrospirota and Actinomycetota, respectively), and nitric oxide reductase (*norC*), were also reduced upon watering. Conversely, peptide transporter transcripts (*livKH, dppA*) significantly increased in response to water addition for several taxa (Actinomycetota, Alpha-, Beta-, and Delta-proteobacteria), suggesting that organic nitrogen dominated N acquisition processes.

Changes in phosphorus acquisition pathways were also identified. Phosphate acquisition after water addition was dominated by the proteobacterial *ugpB* gene from the *sn*-glycerol 3-phosphate (G3P) transport system, particularly in Alpha-proteobacteria, followed by the *pst* inorganic phosphate transporter (Fig. 5B). By contrast, phosphonate transport (*phn*) transcription was reduced after soil wetting.

### Analysis of viral transcripts

A total of 68 contigs were characterized as being of viral origin using VirSorter (Figure S5, Supporting Information). In both dry and watered soil samples, viral contigs were significantly lower than bacterial contigs (ANOVA, *P* < .01), representing an average of 0.12 ± 0.07% TPM of viral protein transcripts. Read numbers remained low in all dry soil samples. An initial increase in viral RNAs (∼2.2-fold at 10 min) was followed by a 12-fold decline over 7 h (Figure S4, Supporting Information). A second increase of viral read numbers (∼6-fold) occurred between 7 h and 7 days.

To investigate the diversity of the ‘active’ viral population, we used a genome-based network analysis of the shared protein content with the prokaryotic viral genomes (RefSeq v85). This analysis grouped 35 viral contigs into viral clusters (VCs) (Figure S5, Supporting Information). In the network, 10 VCs containing viral contigs from our study were predicted, seven of which did not belong to VCs with RefSeq virus genomes but instead clustered together into novel VCs, and three of which could be assigned taxonomy at the family level (Figure S5, Supporting Information) as members of the *Caudovirales* (*Siphoviridae* and *Leviviridae*).

## Discussion

Studies of the microbial ecology of desert edaphic niches have tended to focus on biological ‘hotspots’: hypoliths, soil crusts, and soils in the immediate vicinity of plants (e.g. Pointing and Belnap 2012, Marasco et al. 2018, Ramond et al. 2018). While the use of high-throughput sequencing techniques (Crits-Christoph et al. 2013, Fierer et al. 2007, 2012, Jordaan et al. 2020, Vikram et al. 2016, Marasco et al. 2022) has greatly expanded of knowledge of the microbial ecology of desert soil niches, most studies have used metagenomics. Total RNA sequencing (metatranscriptomics) is, therefore, a valuable tool for monitoring microbial functionality at a high temporal resolution (Moran et al. 2013), particularly since mRNA is only generated by active organisms and is ephemeral, leaving little to no legacy signal (León-Sobrino et al. 2019, Rajeev et al. 2013, Steven et al. 2018).

Previous studies have demonstrated long-term compositional and functional adaptation of Namib Desert edaphic and hypolithic microbial communities to abiotic factors, particularly water input regime histories (e.g. fog vs. rain inputs; Cowan et al. 2020, Frossard et al. 2015, Ramond et al. 2018, Scola et al. 2018, Stomeo et al. 2013, Vikram et al. 2023).

Water events in deserts may trigger different biological responses depending on their intensity and duration (Schwinning and Sala 2004, Frossard et al. 2015). Whereas certain processes respond to small moisture events, especially on the immediate surface of soils and rocks, other ecological responses and niches require a larger pulse to be activated (Pointing and Belnap 2012, Schwinning and Sala 2004). Grass germination, for example, is triggered after around 20 mm rainfall (Seely and Pallett 2008). For this work, focused on subsurface soils, we chose the central hyperarid zone of the Namib Desert, which receives water approximately equally from rain and fog (Eckardt et al. 2013). This ‘neutral’ location would optimize the responsiveness of the soil microbiome to the water input (Frossard et al. 2015) and serve as a baseline reference for either rain- or fog-shaped soil microbiomes. We decided to saturate the soil with 30 l/m^2^of water to ensure a complete biological activation and homogeneous sample conditions in the first centimetres of soil, rather than an arbitrary point along the gradient of possible precipitation events. Although the most frequent rains in deserts are < 5 mm (Pointing and Belnap 2012), heavy rain events > 20 mm also occur in nature (Eckardt et al. 2013, Armstrong et al. 2016).

### The HSP20 chaperone is important for microbial life in desiccated soils

One of the projected effects of water addition was an apparent reduction in cellular stress as suggested by the immediate downregulation in transcription of stress-resistance genes, such as trehalose biosynthesis genes or chaperones. The most conspicuous of these changes was the abrupt reduction in transcription, across all major bacterial taxa, of the small ATP-independent heat-shock protein HSP20. This chaperone has been characterized as a broad-spectrum bacterial stress resistance mechanism (Bepperling et al. 2012, Haslbeck and Vierling 2015). Functional characterization of this protein remains limited, but our data suggest that this protein is specifically involved in desiccation stress adaptation in many bacteria.

### Desert soil microbes are sequentially activated after a water event

Data from the control (unwatered) soil site confirmed the presence of a diverse and functionally active microbial community in desiccated hyperarid desert soils (Gunnigle et al. 2017, Jordaan et al. 2020, León-Sobrino et al. 2019, Schulze-Makuch et al. 2018). Our data also suggest a level of remarkable ‘metabolic readiness’, with a dramatic increase in transcription associated with previously inactive or undetected taxa occurred within 10 min after water addition. We note that transcriptional response rates may be even faster, given that 10 min was the first sampling time-point. In polyextreme hyperarid desert soils, where most microorganisms remain in a state of metabolic dormancy (Bär et al. 2002, Lebre et al. 2017), such a rapid and opportunistic response to the sudden availability of water is clearly an adaptative advantage to access and utilize more favourable, and newly available, ecological substrates and niches.

Water addition led to a general increase in the relative abundance of ribosomal protein transcripts (Rp: T), which we interpret as an increase in cellular activity (Bosdriesz et al. 2015, Bremer and Dennis 1996). Cellular activity levels returned to basal (control) levels within 7 days, in parallel with the desiccation of the soil samples. The characteristic temporal patterns after watering and contrasting stability of control samples suggest that Rp: T is indeed a regulated factor in bacterial cells, supporting its use as a global activity indicator. Our data are consistent with the paradigm that desert ecosystems and their indigenous microbiota are both resilient and water-pulse responsive (e.g. Belnap et al. 2005, Noy-Meir 1973, Armstrong et al. 2016).

Interestingly, various groups of taxa reached maximum Rp: T-values at different times, suggesting a controlled pattern of functionality reminiscent of a stepwise model where ecosystem functions gradually evolve as a function of the duration and intensity of the water pulses (Schwinning and Sala 2004, Placella et al. 2012). The most immediate microbial response was characterized by transcription of genes implicated in the motility apparatus (type IV pili in Alpha-proteobacteria and flagella in Actinomycetota). Accordingly, chemotaxis genes from the same taxa were upregulated, although in a less dramatic manner. It has been previously noted that one of the main impacts of water inundation is increased soil particle connectivity, providing access to new niches and solubilized nutrients (Schimel 2018). Actinomycetota and Alpha-proteobacteria have been reported as the dominant active taxa in desiccated soils (León-Sobrino et al. 2019), possibly uniquely positioned to access new and more favourable niches during periods of interconnection associated with the water-saturated state.

A significant, but delayed, transcriptional activation was observed in the nonfungal microbial eukaryotes (e.g. protists) and Delta-proteobacteria; i.e. 3–7 h after water addition. The former were mostly characterized by structural gene transcripts from the cytoskeleton, a generic indication of overall cellular activity (cell motility and/or cell division). Upregulated Delta-proteobacterial transcripts were predominantly derived from the Myxococcales, an order of well-known predatory bacteria (Jurkevitch and Davidov 2007, Shimkets et al. 2006). The dramatic increase in protist and myxobacterial activity is strongly suggestive of predatory behaviour (Thiery and Kaimer 2020), possibly triggered by increases in prey abundance (i.e. Actinomycetota and Alphaproteobacteria populations) rather than just by soil rehydration.

This study provides, to our knowledge, the first temporal, rather than spatial, assessment of phages in a desert edaphic environment (Hwang et al. 2021, Zablocki et al. 2016, 2017). In parallel with the rapid changes in bacterial metatranscriptomic patterns, the phage population responded within 10 min after water addition, followed by a sharp decrease. We hypothesize that, mirroring the initial burst of motility in some microbial taxa, there might be a readiness for the rapid generation of virions and expansion to new hosts.

### Fungal and cyanobacterial soil populations are not significantly activated by water

Among the edaphic taxa that were essentially nonresponsive to water addition, we particularly identified the Cyanobacteria and most fungi, with almost none of their genes being differentially upregulated at a statistically significant level.

This is a surprising result: we anticipated that water addition would trigger a significant and rapid increase in primary production markers linked to cyanobacterial and photosynthetic activity, as previously observed in BSCs and hypolithic communities (Angel and Conrad 2013, Oren et al. 2017, Pringault and Garcia-Pichel 2004, Rajeev et al. 2013, Steven et al. 2018, Warren-Rhodes et al. 2006). The almost complete absence of water input-related activation of cyanobacterial functionality suggests that primary productivity in hyperarid soils may not be driven by cyanobacteria and is consistent with previous observations showing that hypolithons (and maybe other cryptic communities) are the foundation of productivity after rain events in the Namib Desert (Ramond et al. 2018).

### Water pulses shift microbial C, N, and P nutrient utilization patterns

A peak of respiration during water-triggered blooms in a well-known phenomenon (Austin et al. 2004, Placella et al. 2012). We also expected a significant increase in primary productivity, since photosynthetic processes are highly sensitive to water (e.g. Brock 1975, Steven et al. 2018, Warren-Rhodes et al. 2006). However, markers for photosynthetic and chemoautotrophic carbon fixation (the latter being active in desiccated periods; León-Sobrino et al. 2019, Sghaier et al. 2016), were either not activated or significantly suppressed. Thus, the carbon balance during these water pulses appears to be almost entirely negative in the bulk soil. Our measurements of the organic carbon content of soils indicated very low amounts well below 0.1% wt. (Table S1, Supporting Information), to fuel this activity bloom. Open soil communities may be dependent on carbon input from alternative sources, such as sporadic vegetation growth, productive cryptic niches such as hypolithons and BSCs (Armstrong et al. 2016, Ramond et al. 2018) and/or little-known autotrophic processes such as trace gas chemotrophy (e.g. Greening and Grinter 2022, Jordaan et al. 2020).

Nitrogen cycling genes, particularly those involved in inorganic nitrogen acquisition (i.e. nitrate, via nitrate reductases), were not significantly upregulated after water addition. We note that compositional effects—since we measure relative, rather, than absolute, abundances—might be responsible for the apparent reduction in N cycling transcripts. This was nonetheless surprising, as active N mineralization, nitrification and N loss processes are often increased in arid soils in response to rainfall (Austin et al. 2004, Belnap et al. 2005, Ramond et al. 2022), due to both biological activity and solubilization of nitrate, forming substantial reserves of underground N essentially in the form of nitrate (Graham et al. 2008, Walvoord 2003). A metabolic switch to nitrogen acquisition from organic substrates was strongly suggested by the upregulation of peptide transporter genes, mirroring the situation observed for carbon acquisition. It is most probably linked to the important release of N-rich compounds (e.g. proteins) following the intense death of soil microbial biomass via osmolysis (i.e. between a third to half of it; Belnap et al. 2005). The transient reduction in autotrophic C and N fixation after watering may be explained in terms of energy efficiency, where the sudden availability of ‘energy-rich’ substrates provides a favoured heterotrophic resource over energetically expensive autotrophic processes (Fuchs 2011).

The addition of water triggered an upregulation of genes involved in inorganic phosphate transport and a downregulation of those implicated in organic phosphonate acquisition. We speculate that the solubilization of inorganic phosphate from soil particles displaces phosphonates as the preferred P source (Schowanek and Verstraete 1990). Noticeable exceptions were the Alpha-proteobacterial taxa, which apparently favour organic G3P as a preferred P source, both in desiccated soils and after wetting (León-Sobrino et al. 2019).

### A conceptual response model of desert soil edaphic microbial communities to water

**Figure 6.**
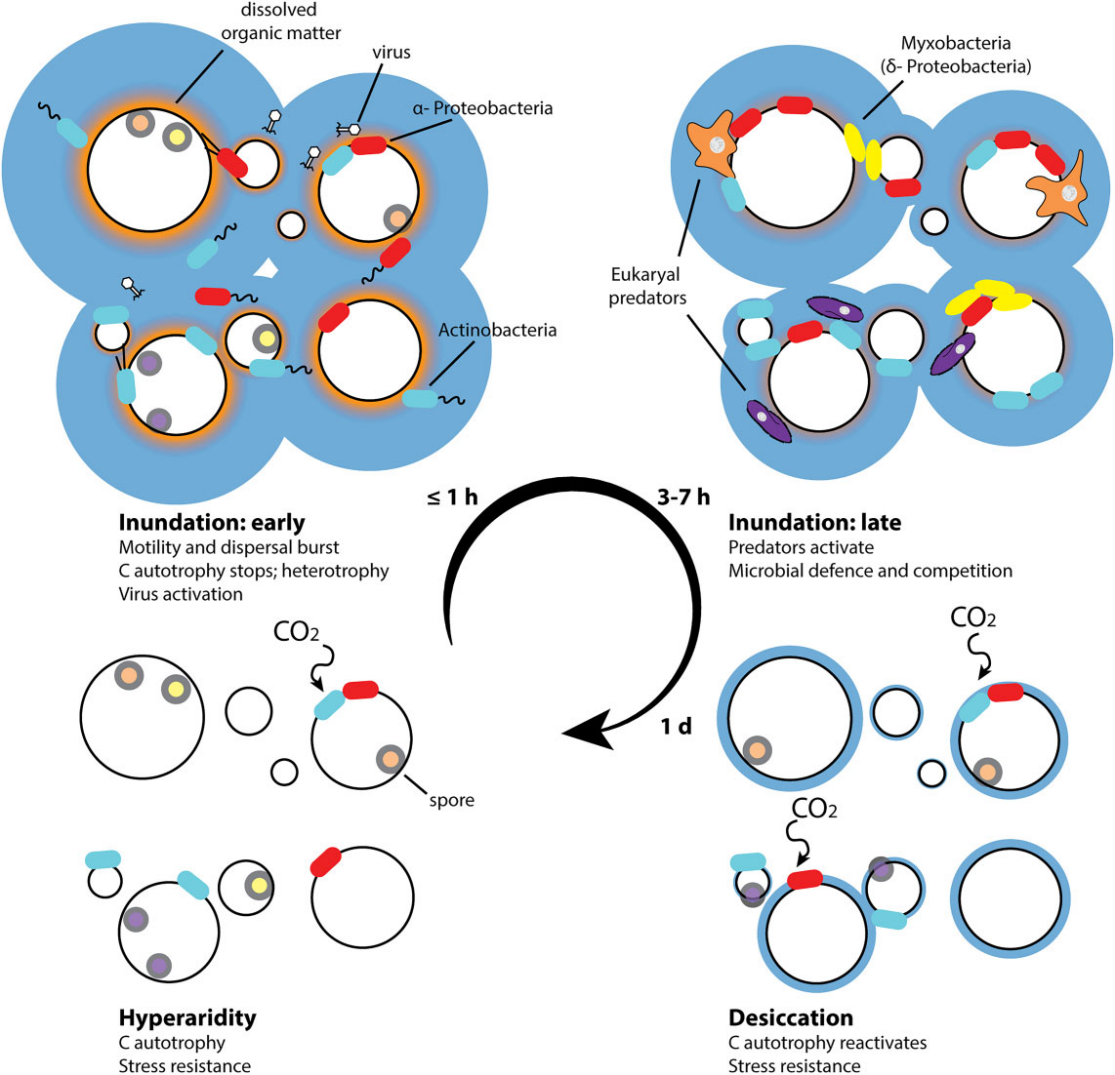
Response model of microbial communities to water events in hyperarid desert soils.

From a composite analysis of our metatranscriptomic data, we propose a rainfall response model for desert soil microbiomes (Fig. 6). Immediately after soil wetting (≤ 10 min), some bacterial taxa (particularly Actinomycetota and Alpha-proteobacteria, that show significant chemoautotrophic capacity in dry soils; León-Sobrino et al. 2019) reduce autotrophic carbon fixation activities, activate cellular uptake mechanisms and engage in dispersal using both natatory (flagella) and gliding (type IV pili) mechanisms, presumably in order to colonize new niches and access new substrate resource pools. There is a concomitant increase in active phage particles, which may add to the turnover of organic matter and the predatory pressure on microbial populations upon the initial rapid wave of dispersal.

Following this rapid dispersal burst, ∼3–7 h after water addition, predatory and saprophytic microbial taxa are activated. These predators include eukaryotes, especially ciliates (Oligohymenophorea class), Dictyostellid amoebae, Delta-proteobacteria (myxobacteria), and Bacteroidota (Cytophagia class). Simultaneously, and presumably in response to the activation of predators, several bacterial groups upregulate the transcription of defensive systems, most notably T6SS.

In the final stage of the wetting–drying cycle (∼7 days after water addition), when soils are effectively dehydrated to prewatering levels, autotrophic carbon fixation processes and nitrogen cycling are reactivated in the bacterial community, along with certain desiccation stress resistance mechanisms, most especially HSP20.

## Conclusion

In this 7-day *in situ* metatranscriptomics study, we profiled the dominant microbial processes induced by precipitation on a hyperarid desert soil. Overall, RNA analysis proved to be a robust tool for microbiome profiling from low biomass environmental samples. The dynamic and function-targeted nature of this mRNA-dependent analyses allowed us to capture short-term variations in microbiome structure and function and offers a valuable complementary analysis tool for environmental microbiomics.

The transcriptomes described a cyclical pattern of community functionality starting immediately after water addition, and returning to the basal state following soil drying. We show robust evidence of short-term temporal succession and, by implication, tightly regulated processes. Shared functional responses across taxa suggest that some functions are important for adaptation to these ecosystems. In particular, we observed a dispersion–predation dynamic and a strong shift towards a heterotrophic lifestyle upon watering. Some characteristic ‘dry genes’ were also documented, particularly those involved in chemoautotrophic carbon fixation (in accordance with previous reports by León-Sobrino et al. 2019), and also HSP20, which might be a key chaperone for adaptation to desiccation stress in many bacterial taxa. These function-over-taxonomy observations may be conserved in microbiomes from other locations sharing similar environmental conditions, and we envision that this study can serve to inspire future work in that direction.

## Supplementary Material

fiae009_Supplemental_File

## Data Availability

The dataset supporting the conclusions of this article is available in the ArrayExpress repository, https://www.ebi.ac.uk/arrayexpress/ (accession number E-MTAB-9439). Reference metatranscriptome assembly and annotations can be accessed at the IMG/M repository, https://img.jgi.doe.gov/ (GOLD Analysis Project Id Ga0326365.)

## References

[bib2] Angel R, Conrad R. Elucidating the microbial resuscitation cascade in biological soil crusts following a simulated rain event. Environ Microbiol. 2013;15:2799–815. 10.1111/1462-2920.12140. 23648088

[bib3] Armstrong A, Valverde A, Ramond JB et al. Temporal dynamics of hot desert microbial communities reveal structural and functional responses to water input. Sci Rep. 2016;6:34434. 10.1038/srep34434. 27680878 PMC5041089

[bib4] Austin AT, Yahdjian L, Stark JM et al. Water pulses and biogeochemical cycles in arid and semiarid ecosystems. Oecologia. 2004;141:221–35. 10.1007/s00442-004-1519-1. 14986096

[bib5] Bär M, Hardenberg J, Meron E et al. Modelling the survival of bacteria in drylands: the advantage of being dormant. Proc R Soc Lond Ser B Biol Sci. 2002;269:937–42. 10.1098/rspb.2002.1958. PMC1690970 12028777

[bib6] bbmap . Masked version of hG19 by Brian Bushnell. Zenodo, 2018. 10.5281/ZENODO.1208052.

[bib7] Belnap J, Welter JR, Grimm NB et al. Linkages between microbial and hydrologic processes in arid and semiarid watersheds. Ecology. 2005;86:298–307. 10.1890/03-0567.

[bib8] Bepperling A, Alte F, Kriehuber T et al. Alternative bacterial two-component small heat shock protein systems. Proc Natl Acad Sci USA. 2012;109:20407–12. 10.1073/pnas.1209565109. 23184973 PMC3528540

[bib10] Bin Jang H, Bolduc B, Zablocki O et al. Taxonomic assignment of uncultivated prokaryotic virus genomes is enabled by gene-sharing networks. Nat Biotechnol. 2019;37:632–9. 10.1038/s41587-019-0100-8. 31061483

[bib11] Bolduc B, Youens-Clark K, Roux S et al. IVirus: facilitating new insights in viral ecology with software and community data sets imbedded in a cyberinfrastructure. ISME J. 2017;11:7–14. 10.1038/ismej.2016.89. 27420028 PMC5315481

[bib12] Boratyn GM, Schäffer AA, Agarwala R et al. Domain enhanced lookup time accelerated BLAST. Biol Direct. 2012;7:12. 10.1186/1745-6150-7-12. 22510480 PMC3438057

[bib13] Bosdriesz E, Molenaar D, Teusink B et al. How fast-growing bacteria robustly tune their ribosome concentration to approximate growth-rate maximization. FEBS J. 2015;282:2029–44. 10.1111/febs.13258. 25754869 PMC4672707

[bib14] Bremer H, Dennis P. Modulation of chemical composition and other parameters of the cell by growth rate. EcoSal Plus. 1996;2. 10.1128/ecosal.5.2.3. 26443740

[bib15] Brock TDT . Effect of water potential on a *Microcoleus* (Cyanophyceae) from a desert crust. J Phycol. 1975;11:316–20. 10.1111/j.1529-8817.1975.tb02786.x.

[bib16] Bushnell B, Rood J, Singer E. BBMerge—accurate paired shotgun read merging via overlap. PLoS ONE. 2017;12:e0185056. 10.1371/journal.pone.0185056. 29073143 PMC5657622

[bib17] Collins SL, Belnap J, Grimm NB et al. A multiscale, hierarchical model of pulse dynamics in arid-land ecosystems. Annu Rev Ecol Evol Syst. 2014;45:397–419. 10.1146/annurev-ecolsys-120213-091650.

[bib18] Cowan DA, Hopkins DW, Jones BE et al. Microbiomics of Namib Desert habitats. Extremophiles. 2020;24:17–29. 10.1007/s00792-019-01122-7. 31376000

[bib19] Crits-Christoph A, Robinson CK, Barnum T et al. Colonization patterns of soil microbial communities in the Atacama Desert. Microbiome. 2013;1:28. 10.1186/2049-2618-1-28. 24451153 PMC3971613

[bib20] Delgado-Baquerizo M, Maestre FT, Gallardo A et al. Decoupling of soil nutrient cycles as a function of aridity in global drylands. Nature. 2013;502:672–6. 10.1038/nature12670. 24172979

[bib21] Delgado-Baquerizo M, Maestre FT, Reich PB et al. Microbial diversity drives multifunctionality in terrestrial ecosystems. Nat Commun. 2016;7:10541. 10.1038/ncomms10541. 26817514 PMC4738359

[bib22] Eckardt FD, Soderberg K, Coop LJ et al. The nature of moisture at Gobabeb, in the central Namib Desert. J Arid Environ. 2013;93:7–19. 10.1016/j.jaridenv.2012.01.011.

[bib23] Fierer N, Breitbart M, Nulton J et al. Metagenomic and small-subunit rRNA analyses reveal the genetic diversity of bacteria, archaea, fungi, and viruses in soil. Appl Environ Microb. 2007;73:7059–66. 10.1128/AEM.00358-07. PMC2074941 17827313

[bib24] Fierer N, Leff JW, Adams BJ et al. Cross-biome metagenomic analyses of soil microbial communities and their functional attributes. Proc Natl Acad Sci USA. 2012;109:21390–5. 10.1073/pnas.1215210110. 23236140 PMC3535587

[bib25] Frossard A, Ramond J-B, Seely M et al. Water regime history drives responses of soil Namib Desert microbial communities to wetting events. Sci Rep. 2015;5:12263. 10.1038/srep12263. 26195343 PMC4508562

[bib26] Fuchs G . Alternative pathways of carbon dioxide fixation: insights into the early evolution of life?. Annu Rev Microbiol. 2011;65:631–58. 10.1146/annurev-micro-090110-102801. 21740227

[bib27] Garcia-Pichel F, Pringault O. Cyanobacteria track water in desert soils. Nature. 2001;413:380–1. 10.1038/35096640. 11574875

[bib28] Gee GW, Bauder JW. 1986. Particle-size analysis. In: Klute A (ed.), Methods of Soil Analysis: Part 1—Physical and Mineralogical Methods, SSSA Book Series SV–5.1. Madison: Soil Science Society of America, American Society of Agronomy, 383–411. 10.2136/sssabookser5.1.2ed.c15.

[bib29] Gombeer S, Ramond J-B, Eckardt FD et al. The influence of surface soil physicochemistry on the edaphic bacterial communities in contrasting terrain types of the Central Namib Desert. Geobiology. 2015;13:494–505. 10.1111/gbi.12144. 25939371

[bib30] Graham RC, Hirmas DR, Wood YA et al. Large near-surface nitrate pools in soils capped by desert pavement in the Mojave Desert, California. Geology. 2008;36:259–62. 10.1130/G24343A.1.

[bib31] Greening C, Grinter R. Microbial oxidation of atmospheric trace gases. Nat Rev Microbiol. 2022;20:513–28. 35414013 10.1038/s41579-022-00724-x

[bib32] Gunnigle E, Frossard A, Ramond J-B et al. Diel-scale temporal dynamics recorded for bacterial groups in Namib Desert soil. Sci Rep. 2017;7:40189. 10.1038/srep40189. 28071697 PMC5223211

[bib33] Gwizdala M, Lebre PH, Maggs-Kölling G et al. Sub-lithic photosynthesis in hot desert habitats. Environ Microbiol. 2021;23:3867–80. 10.1111/1462-2920.15505. 33817951

[bib34] Haslbeck M, Vierling E. A first line of stress defense: small heat shock proteins and their function in protein homeostasis. J Mol Biol. 2015;427:1537–48. 10.1016/J.JMB.2015.02.002. 25681016 PMC4360138

[bib35] Huang J, Yu H, Guan X et al. Accelerated dryland expansion under climate change. Nat Clim Chang. 2016;6:166–71. 10.1038/nclimate2837.

[bib36] Huntemann M, Ivanova NN, Mavromatis K et al. The standard operating procedure of the DOE-JGI Microbial Genome Annotation Pipeline (MGAP v.4). Stand Genomic Sci. 2015;10:86. 10.1186/s40793-015-0077-y. 26512311 PMC4623924

[bib37] Hwang Y, Rahlff J, Schulze-Makuch D et al. Diverse viruses carrying genes for microbial extremotolerance in the Atacama Desert hyperarid soil. mSystems. 2021;6:e00385–21. 34006626 10.1128/mSystems.00385-21 PMC8269230

[bib38] Hyatt D, Chen G-L, Locascio PF et al. Prodigal: prokaryotic gene recognition and translation initiation site identification. BMC Bioinf. 2010;11:119. 10.1186/1471-2105-11-119. PMC2848648 20211023

[bib41] Jordaan K, Lappan R, Dong X et al. Hydrogen-oxidizing bacteria are abundant in desert soils and strongly stimulated by hydration. mSystems. 2020;5:e01131–20. 10.1128/mSystems.01131-20. 33203691 PMC7677003

[bib42] Jurkevitch E, Davidov Y. Phylogenetic diversity and evolution of predatory prokaryotes. In: Predatory Prokaryotes. Microbiology Monographs. Berlin: Springer. 2007, 11–56. 10.1007/7171_052.

[bib43] Kanehisa M, Sato Y, Kawashima M et al. KEGG as a reference resource for gene and protein annotation. Nucleic Acids Res. 2016;44:D457–62. 10.1093/nar/gkv1070. 26476454 PMC4702792

[bib44] Laity JJ . Deserts and Desert Environments. Vol. 3, Chichester: Wiley-Blackwell, 2009.

[bib45] Langmead B, Salzberg SL. Fast gapped-read alignment with Bowtie 2. Nat Methods. 2012;9:357–9. 10.1038/nmeth.1923. 22388286 PMC3322381

[bib46] Lebre PH, De Maayer P, Cowan DA. Xerotolerant bacteria: surviving through a dry spell. Nat Rev Micro. 2017;15:285–96. 10.1038/nrmicro.2017.16. 28316329

[bib47] Lennon JT, Muscarella ME, Placella SA et al. How, when, and where relic DNA affects microbial diversity. mBio. 2018;9:e00637–18. 29921664 10.1128/mBio.00637-18 PMC6016248

[bib48] León-Sobrino C, Ramond J-BBJ-B, Maggs-Kölling G et al. Nutrient acquisition, rather than stress response over diel cycles, drives microbial transcription in a hyper-arid Namib Desert soil. Front Microbiol. 2019;10:1054. 10.3389/fmicb.2019.01054. 31139170 PMC6527771

[bib49] Li H, Handsaker B, Wysoker A et al. The sequence alignment/map format and SAMtools. Bioinformatics. 2009;25:2078–9. 10.1093/bioinformatics/btp352. 19505943 PMC2723002

[bib50] Liao Y, Smyth GK, Shi W. FeatureCounts: an efficient general purpose program for assigning sequence reads to genomic features. Bioinformatics. 2014;30:923–30. 10.1093/bioinformatics/btt656. 24227677

[bib51] Love MI, Huber W, Anders S. Moderated estimation of fold change and dispersion for RNA-seq data with DESeq2. Genome Biol. 2014;15:550. 10.1186/s13059-014-0550-8. 25516281 PMC4302049

[bib53] Maestre FT, Delgado-Baquerizo M, Jeffries TC et al. Increasing aridity reduces soil microbial diversity and abundance in global drylands. Proc Natl Acad Sci USA. 2015;112:15684–9. 10.1073/pnas.1516684112. 26647180 PMC4697385

[bib55] Marasco R, Fusi M, Ramond JB et al. The plant rhizosheath–root niche is an edaphic “mini-oasis” in hyperarid deserts with enhanced microbial competition. ISME Commun. 2022;2:47. 37938683 10.1038/s43705-022-00130-7 PMC9723607

[bib56] Marasco R, Mosqueira MJ, Fusi M et al. Rhizosheath microbial community assembly of sympatric desert speargrasses is independent of the plant host. Microbiome. 2018;6:215. 10.1186/s40168-018-0597-y. 30514367 PMC6280439

[bib57] Marchler-Bauer A, Derbyshire MK, Gonzales NR et al. CDD: NCBI's Conserved Domain Database. Nucleic Acids Res. 2015;43:D222–6. 10.1093/nar/gku1221. 25414356 PMC4383992

[bib59] Moran MA, Satinsky B, Gifford SM et al. Sizing up metatranscriptomics. ISME J. 2013;7:237–43. 10.1038/ismej.2012.94. 22931831 PMC3554401

[bib100_961_154424] Noy-Meir I . Desert ecosystems: environment and producers. Annu Rev Ecol Syst. 1973;4:25–51.

[bib60] Oren N, Raanan H, Murik O et al. Dawn illumination prepares desert cyanobacteria for dehydration. Curr Biol. 2017;27:R1056–7. 10.1016/j.cub.2017.08.027. 29017037

[bib62] Placella SA, Brodie EL, Firestone MK. Rainfall-induced carbon dioxide pulses result from sequential resuscitation of phylogenetically clustered microbial groups. Proc Natl Acad Sci USA. 2012;109:10931–6. 10.1073/pnas.1204306109. 22715291 PMC3390866

[bib63] Pointing SBBB, Belnap J. Microbial colonization and controls in dryland systems. Nat Rev Micro. 2012;10:551–62. 10.1038/nrmicro2831. 22772903

[bib64] Pringault O, Garcia-Pichel F. Hydrotaxis of cyanobacteria in desert crusts. Microb Ecol. 2004;47:366–73. 10.1007/s00248-002-0107-3. 14605777

[bib65] Quast C, Pruesse E, Yilmaz P et al. The SILVA ribosomal RNA gene database project: improved data processing and web-based tools. Nucleic Acids Res. 2012;41:D590–6. 10.1093/nar/gks1219. 23193283 PMC3531112

[bib66] Rajeev L, Da Rocha UNN, Klitgord N et al. Dynamic cyanobacterial response to hydration and dehydration in a desert biological soil crust. ISME J. 2013;7:2178–91. 10.1038/ismej.2013.83. 23739051 PMC3806265

[bib67] Ramond J-B, Jordaan K, Díez B et al. Microbial biogeochemical cycling of nitrogen in arid ecosystems. Microbiol Mol Biol Rev. 2022;86:e00109–21. 10.1128/mmbr.00109-21. 35389249 PMC9199420

[bib68] Ramond J-B, Woodborne S, Hall G et al. Namib Desert primary productivity is driven by cryptic microbial community N-fixation. Sci Rep. 2018;8:6921. 10.1038/s41598-018-25078-4. 29720684 PMC5932006

[bib69] Robertson G, Schein J, Chiu R et al. *De novo* assembly and analysis of RNA-seq data. Nat Methods. 2010;7:909–12. 10.1038/nmeth.1517. 20935650

[bib72] Roux S, Enault F, Hurwitz BL et al. VirSorter: mining viral signal from microbial genomic data. PeerJ. 2015;2015:1–20. 10.7717/peerj.985. PMC4451026 26038737

[bib73] Schimel JP . Life in dry soils: effects of drought on soil microbial communities and processes. Annu Rev Ecol Evol Syst. 2018;49:409–32. 10.1146/annurev-ecolsys-110617-062614.

[bib74] Schlesinger WH, Raikes JA, Hartley AE et al. On the spatial pattern of soil nutrients in desert ecosystems. Ecology. 1995;77:364–74. 10.2307/2265615.

[bib75] Schowanek D, Verstraete W. Phosphonate utilization by bacteria in the presence of alternative phosphorus sources. Biodegradation. 1990;1:43–53. 10.1007/BF00117050. 1368141

[bib76] Schulze-Makuch D, Wagner D, Kounaves SP et al. Transitory microbial habitat in the hyperarid Atacama Desert. Proc Natl Acad Sci USA. 2018;115:2670–5. 10.1073/pnas.1714341115. 29483268 PMC5856521

[bib77] Schwinning S, Sala OE. Hierarchy of responses to resource pulses in arid and semi-arid ecosystems. Oecologia. 2004;141:211–20. 10.1007/s00442-004-1520-8. 15034778

[bib78] Scola V, Ramond J-B, Frossard A et al. Namib Desert soil microbial community diversity, assembly, and function along a natural xeric gradient. Microb Ecol. 2018;75:193–203. 10.1007/s00248-017-1009-8. 28647755

[bib79] Seely M, Pallett J, Namib: Secrets of a Desert Uncovered, Venture. Windhoek: Venture Publications, 2008.

[bib80] Sghaier H, Hezbri K, Ghodhbane-Gtari F et al. Stone-dwelling actinobacteria *Blastococcus saxobsidens, Modestobacter marinus* and *Geodermatophilus obscurus* proteogenomes. ISME J. 2016;10:21–9. 10.1038/ismej.2015.108. 26125681 PMC4681853

[bib81] Shimkets L, Dworkin M, Reichenbach H. The myxobacteria BT. In: Dworkin M, Falkow S, Rosenberg E et al. et al. (eds.), The Prokaryotes. Proteobacteria: Delta and Epsilon Subclasses. Deeply Rooting. Vol. 7. New York: Springer, 2006, 31–115.

[bib82] Smoot ME, Ono K, Ruscheinski J et al. Cytoscape 2.8: new features for data integration and network visualization. Bioinformatics. 2011;27:431–2. 10.1093/bioinformatics/btq675. 21149340 PMC3031041

[bib83] Steven B, Belnap J, Kuske CR. Chronic physical disturbance substantially alters the response of biological soil crusts to a wetting pulse, as characterized by metatranscriptomic sequencing. Front Microbiol. 2018;9:2382. 10.3389/fmicb.2018.02382. 30349515 PMC6186815

[bib84] Stomeo F, Valverde A, Pointing SB et al. Hypolithic and soil microbial community assembly along an aridity gradient in the Namib Desert. Extremophiles. 2013;17:329–37. 10.1007/s00792-013-0519-7. 23397517

[bib85] Štovíček A, Kim M, Or D et al. Microbial community response to hydration-desiccation cycles in desert soil. Sci Rep. 2017;7:45735. 10.1038/srep45735. 28383531 PMC5382909

[bib86] Szymanski M, Zielezinski A, Barciszewski J et al. 5SRNAdb: an information resource for 5S ribosomal RNAs. Nucleic Acids Res. 2016;44:D180–3. 10.1093/nar/gkv1081. 26490961 PMC4702797

[bib87] Thiery S, Kaimer C. The predation strategy of *Myxococcus xanthus*. Front Microbiol. 2020;11:2. 10.3389/fmicb.2020.00002. 32010119 PMC6971385

[bib88] Tracy CR, Streten-Joyce C, Dalton R et al. Microclimate and limits to photosynthesis in a diverse community of hypolithic cyanobacteria in northern Australia. Environ Microbiol. 2010;12:592–607. 10.1111/j.1462-2920.2009.02098.x. 19919538

[bib92] Vikram S, Guerrero LD, Makhalanyane TP et al. Metagenomic analysis provides insights into functional capacity in a hyperarid desert soil niche community. Environ Microbiol. 2016;18:1875–88. 10.1111/1462-2920.13088. 26470632

[bib93] Vikram S, Ramond J-B, Ortiz M et al. Soil fungal diversity and assembly along a xeric stress gradient in the central Namib Desert. Fung Biol. 2023;127:997–1003. 10.1016/j.funbio.2023.03.001. 37024159

[bib94] Walkley A . An examination of methods for determining organic carbon and nitrogen in soils. J Agric Sci. 1935;25:598–609. 10.1017/S0021859600019687.

[bib95] Walvoord MA . A reservoir of nitrate beneath desert soils. Science. 2003;302:1021–4. 10.1126/science.1086435. 14605364

[bib96] Warren-Rhodes KA, Rhodes KL, Pointing SB et al. Hypolithic cyanobacteria, dry limit of photosynthesis, and microbial ecology in the hyperarid Atacama Desert. Microb Ecol. 2006;52:389–98. 10.1007/s00248-006-9055-7. 16865610

[bib98] Zablocki O, Adriaenssens EM, Cowan D. Diversity and ecology of viruses in hyperarid desert soils. Appl Environ Microb. 2016;82:770–7. 10.1128/AEM.02651-15. PMC4725269 26590289

[bib99] Zablocki O, Adriaenssens EM, Frossard A et al. Metaviromes of extracellular soil viruses along a Namib Desert aridity gradient. Genome Announc. 2017;5:4–5. 10.1128/genomeA.01470-16. PMC5256219 28082503

