# Supplementary material for: Temporal dynamics of microbial transcription in wetted hyperarid desert soils
Source: FEMS Microbiol Ecol. 2024 Jan 31;100(3):fiae009. doi: 10.1093/femsec/fiae009 (PMC10913055; doi:10.1093/femsec/fiae009)
Supplement: fiae009_Supplemental_File [file fiae009_supplemental_file.docx]

# Supplementary Information

**Temporal dynamics of microbial transcription in wetted hyperarid desert soils**

Carlos León-Sobrino^1,2^, Jean-Baptiste Ramond^1,3^, Clément Coclet^1^, Ritha-Meriam Kapitango^4^, Gillian Maggs-Kölling^4^, and Don A. Cowan^1, *^

^1^ Centre for Microbial Ecology and Genomics, Department of Biochemistry, Genetics and Microbiology, University of Pretoria, Pretoria, South Africa.

^2^ The Novo Nordisk Foundation Center for Biosustainability, Technical University of Denmark, 2800 Kgs Lyngby, Denmark.

^3^ Extreme Ecosystem Microbiomics & Ecogenomics (E²ME) Lab., Facultad de Ciencias Biológicas, Pontificia Universidad Católica de Chile, Santiago, Chile

^4^ Gobabeb - Namib Research Institute, Walvis Bay, Namibia.

.

## Supplementary tables

| **Sector** | **pH** | **Na** | **K** | **Ca** | **Mg** | **NO_3_^-^** | **NH_4_^+^** | **Cl** | **EC** | **P** | **C** | **Sand** | **Clay** | **Silt** | **>1000 µm** | **>500 µm** | **>250 µm** | **>100 µm** | **>53 µm** | **<53 µm** |
| --- | --- | --- | --- | --- | --- | --- | --- | --- | --- | --- | --- | --- | --- | --- | --- | --- | --- | --- | --- | --- |
|  |  | **mg/l** | **mg/l** | **mg/l** | **mg/l** | **mg/l** | **mg/l** | **mg/l** | **mS/m** | **mg/l** | **%** | **%** | **%** | **%** | **%** | **%** | **%** | **%** | **%** | **%** |
| 10 min C2 | 7.5 | 5.26 | 2 | 2.6 | 1.03 | 0.3 | 0.1 | 9.7 | 8 | 0.19 | 0.03 | 95 | 6 | - | 4.80 | 5.15 | 5.90 | 45.47 | 33.19 | 2.63 |
| 10 min C3 | 7.5 | 5.67 | 2.26 | 3.86 | 1.13 | 0.9 | 1.4 | 11.9 | 9 | 0.2 | 0.06 | 91 | 6 | 3 | 2.48 | 3.64 | 4.43 | 40.21 | 40.47 | 5.28 |
| 10 min W1 | 7.4 | 5.44 | 2.84 | 5.95 | 1.44 | 0.1 | 0.1 | 11.5 | 11 | 0.25 | 0.09 | 91 | 8 | 1 | 4.34 | 5.00 | 4.62 | 41.10 | 35.72 | 2.72 |
| 10 min W2 | 7.5 | 5.15 | 3.04 | 4.17 | 1.37 | 0.7 | 0.7 | 10.8 | 9 | 0.29 | 0.03 | 90 | 6 | 4 | 3.80 | 4.59 | 5.13 | 43.87 | 33.07 | 4.84 |
| 1 h C1 | 7.4 | 5.04 | 2.48 | 2.88 | 1.15 | 0.9 | 2.3 | 12.6 | 8 | 0.2 | 0.03 | 93 | 6 | 1 | 3.44 | 4.05 | 4.71 | 41.70 | 39.51 | 3.23 |
| 1 h C2 | 7.3 | 6.06 | 2.46 | 5.2 | 1.31 | 0.2 | 0.1 | 12.2 | 10 | 0.18 | 0.05 | 93 | 6 | 1 | 3.07 | 3.76 | 5.58 | 47.52 | 33.20 | 4.58 |
| 1 h W2 | 7.1 | 5.89 | 3.23 | 6.73 | 1.45 | 0 | 1.6 | 11.5 | 12 | 0.26 | 0.05 | 91 | 6 | 3 | 3.42 | 3.99 | 4.44 | 42.05 | 37.43 | 5.43 |
| 1 h W3 | 7.2 | 5.08 | 2.97 | 7.44 | 1.51 | 2.6 | 0.6 | 11.6 | 12 | 0.23 | 0.06 | 92 | 6 | 2 | 3.49 | 4.10 | 4.87 | 43.30 | 36.72 | 5.01 |
| 3 h C1 | 7.5 | 5.86 | 2.34 | 3.88 | 1.33 | 0.6 | 1.1 | 11.5 | 9 | 0.22 | 0.08 | 93 | 5 | 2 | 3.49 | 3.95 | 4.67 | 43.15 | 37.59 | 3.97 |
| 3 h C2 | 7.5 | 5.53 | 2.32 | 4.48 | 1.27 | 0.6 | 0.7 | 14.4 | 8 | 0.21 | 0.07 | 92 | 6 | 2 | 3.20 | 3.95 | 4.98 | 45.35 | 34.75 | 4.46 |
| 3 h W1 | 7.3 | 6.12 | 3.12 | 5.39 | 1.48 | 0.8 | 0.1 | 14.4 | 11 | 0.33 | 0.02 | 90 | 6 | 4 | 3.52 | 4.72 | 4.70 | 36.69 | 40.67 | 5.18 |
| 3 h W2 | 7.3 | 5.29 | 2.69 | 4.77 | 1.19 | 0.1 | 0.2 | 10.8 | 10 | 0.29 | 0.03 | 93 | 6 | 1 | 3.44 | 4.27 | 5.13 | 45.48 | 34.38 | 4.31 |
| 7 h C2 | 7.5 | 5.55 | 2.81 | 3.85 | 1.2 | 0.8 | 0.8 | 13 | 10 | 0.19 | 0.05 | 92 | 6 | 2 | 2.55 | 3.27 | 4.23 | 43.11 | 39.05 | 4.73 |
| 7 h C3 | 7.6 | 5.4 | 2.19 | 4.43 | 1.19 | 0.8 | 0.8 | 11.5 | 9 | 0.18 | 0.02 | 94 | 7 | - | 3.99 | 4.01 | 4.74 | 47.69 | 33.81 | 3.56 |
| 7 h W2 | 7.7 | 5.67 | 2.94 | 5.01 | 1.24 | 1.7 | 0.6 | 10.4 | 11 | 0.27 | 0.05 | 91 | 6 | 3 | 3.44 | 4.57 | 4.94 | 45.09 | 33.37 | 4.88 |
| 7 h W3 | 7.5 | 4.87 | 2.7 | 5.41 | 1.31 | 1.1 | 0.6 | 11.2 | 10 | 0.3 | 0.06 | 93 | 6 | 1 | 6.36 | 7.17 | 6.33 | 42.48 | 30.55 | 3.42 |
| 1 d C2 | 7.1 | 5.55 | 2.56 | 3.39 | 1.34 | 1.2 | 0.7 | 9.7 | 8 | 0.25 | 0.04 | 92 | 6 | 2 | 2.64 | 3.36 | 4.44 | 44.30 | 37.51 | 5.13 |
| 1 d C3 | 7.1 | 5.41 | 2.45 | 4.37 | 1.36 | 1 | 1.7 | 12.2 | 8 | 0.22 | 0.02 | 93 | 4 | 3 | 2.82 | 4.69 | 5.82 | 46.84 | 32.72 | 4.43 |
| 1 d W2 | 7.4 | 4.03 | 2.54 | 2.82 | 1.68 | 1 | 1 | 7.2 | 6 | 0.34 | 0.03 | 92 | 6 | 2 | 2.94 | 3.42 | 4.45 | 50.55 | 30.72 | 4.56 |
| 1 d W3 | 7.2 | 4.19 | 2.91 | 2.96 | 1.59 | 0.3 | 1.2 | 8.6 | 7 | 0.42 | 0.04 | 90 | 7 | 3 | 5.04 | 5.68 | 5.64 | 37.62 | 36.17 | 4.63 |
| 7 d C2 | 7.3 | 4.6 | 2.36 | 2.85 | 1.27 | 0.9 | 1.7 | 6.8 | 7 | 0.23 | 0.03 | 93 | 7 | 0 | 2.37 | 3.91 | 5.56 | 46.96 | 34.08 | 3.78 |
| 7 d C3 | 7.5 | 4.98 | 2.49 | 3.61 | 1.27 | 1 | 1.6 | 11.2 | 8 | 0.17 | 0.04 | 92 | 5 | 3 | 2.49 | 3.53 | 4.92 | 44.58 | 36.66 | 4.24 |
| 7 d W2 | 7.5 | 4.23 | 2.04 | 2.66 | 1.19 | 0.6 | 0.9 | 6.8 | 6 | 0.2 | 0.05 | 92 | 6 | 2 | 3.33 | 3.97 | 4.87 | 43.99 | 35.84 | 4.11 |
| 7 d W3 | 8 | 4.3 | 2.56 | 3.24 | 1.46 | 0.7 | 1.3 | 8.6 | 7 | 0.21 | 0.02 | 87 | 8 | 5 | 2.47 | 3.43 | 4.08 | 40.09 | 36.88 | 6.07 |

**Table S1**: Soil composition of samples selected for transcriptome sequencing. C and W in sample identifiers refer to Control (dry) and Watered plots, respectively. All soil properties were determined from particles <2 mm. EC: soil electrical conductivity.

|  |  | **Dry** | | | | | | **Watered** | | | | | | **Dry**  **7-hour ave.** | **Watered 7-hour ave.** |
| --- | --- | --- | --- | --- | --- | --- | --- | --- | --- | --- | --- | --- | --- | --- | --- |
| **Class** | **Phylum** | **10 min** | **1 h** | **3 h** | **7 h** | **1 d** | **7 d** | **10 min** | **1 h** | **3 h** | **7 h** | **1 d** | **7 d** |  |  |
| Actinobacteria | Actinomycetota | 29.66 | 21.82 | 24.11 | 19.60 | 21.99 | 21.24 | 29.51 | 19.53 | 19.52 | 22.18 | 18.83 | 20.28 | 23.80 | 22.68 |
| Rubrobacteria |  | 16.90 | 17.73 | 15.71 | 15.81 | 14.94 | 11.68 | 10.97 | 10.57 | 10.76 | 9.04 | 9.30 | 12.36 | 16.54 | 10.34 |
| Thermoleophilia |  | 4.31 | 3.21 | 3.57 | 3.03 | 2.80 | 3.02 | 5.54 | 4.87 | 4.05 | 2.90 | 3.55 | 4.44 | 3.53 | 4.34 |
| Other |  | 0.66 | 0.72 | 0.61 | 0.81 | 0.86 | 1.86 | 0.94 | 0.91 | 1.03 | 0.66 | 0.66 | 0.67 | 0.70 | 0.88 |
| Total |  | 51.53 | 43.47 | 44.01 | 39.25 | 40.59 | 37.79 | 46.96 | 35.87 | 35.35 | 34.78 | 32.34 | 37.76 | 44.56 | 38.24 |
| Alphaproteobacteria | Pseudomonadota | 9.38 | 10.00 | 12.07 | 9.87 | 9.91 | 9.84 | 12.34 | 9.90 | 13.23 | 12.52 | 8.02 | 5.66 | 10.33 | 12.00 |
| Deltaproteobacteria |  | 1.43 | 1.46 | 1.81 | 1.43 | 2.07 | 3.75 | 3.02 | 3.48 | 5.92 | 6.16 | 3.73 | 1.63 | 1.54 | 4.64 |
| Gammaproteobacteria |  | 1.24 | 1.31 | 1.20 | 1.23 | 1.18 | 1.52 | 1.13 | 1.45 | 1.49 | 1.44 | 1.54 | 1.87 | 1.24 | 1.38 |
| Betaproteobacteria |  | 1.61 | 1.74 | 1.72 | 1.61 | 1.55 | 2.29 | 1.18 | 1.14 | 1.18 | 1.42 | 1.64 | 2.46 | 1.67 | 1.23 |
| Other |  | 0.26 | 0.30 | 0.36 | 0.34 | 0.27 | 0.29 | 0.18 | 0.20 | 0.16 | 0.22 | 0.19 | 0.42 | 0.32 | 0.19 |
| Total |  | 13.92 | 14.81 | 17.15 | 14.49 | 14.97 | 17.69 | 17.84 | 16.16 | 21.97 | 21.77 | 15.12 | 12.03 | 15.09 | 19.44 |
| Nitrososphaeria | Thaumarchaeota | 8.62 | 13.72 | 9.23 | 16.33 | 14.73 | 13.63 | 4.34 | 15.40 | 10.36 | 11.04 | 23.90 | 22.27 | 11.98 | 10.28 |
| Other |  | 0.15 | 0.24 | 0.15 | 0.38 | 0.21 | 0.26 | 0.08 | 0.25 | 0.14 | 0.16 | 0.45 | 0.40 | 0.23 | 0.16 |
| Total |  | 8.76 | 13.96 | 9.38 | 16.71 | 14.94 | 13.89 | 4.42 | 15.65 | 10.50 | 11.20 | 24.36 | 22.67 | 12.20 | 10.44 |
| Chloroflexia | Chloroflexota | 4.30 | 4.32 | 6.24 | 3.95 | 4.58 | 2.87 | 4.37 | 4.06 | 3.87 | 2.42 | 3.12 | 2.39 | 4.70 | 3.68 |
| unclass. Chloroflexi |  | 1.20 | 1.15 | 1.61 | 0.95 | 1.08 | 1.94 | 0.46 | 0.65 | 0.47 | 0.39 | 0.93 | 1.66 | 1.23 | 0.49 |
| Other |  | 4.77 | 4.57 | 5.81 | 4.04 | 3.60 | 3.65 | 3.27 | 3.01 | 2.91 | 1.91 | 2.99 | 4.19 | 4.80 | 2.78 |
| Total |  | 10.27 | 10.05 | 13.66 | 8.94 | 9.26 | 8.46 | 8.09 | 7.72 | 7.26 | 4.72 | 7.04 | 8.24 | 10.73 | 6.95 |
| Bacilli | Bacillota | 1.89 | 2.45 | 2.06 | 3.05 | 3.09 | 5.10 | 1.69 | 2.53 | 2.85 | 2.27 | 2.27 | 2.37 | 2.37 | 2.34 |
| Clostridia |  | 1.01 | 1.27 | 0.97 | 1.01 | 1.34 | 3.59 | 1.46 | 1.62 | 1.53 | 1.11 | 1.22 | 1.36 | 1.06 | 1.43 |
| Other |  | 0.17 | 0.13 | 0.13 | 0.12 | 0.09 | 0.13 | 0.20 | 0.20 | 0.19 | 0.13 | 0.17 | 0.16 | 0.14 | 0.18 |
| Total |  | 3.07 | 3.85 | 3.16 | 4.19 | 4.53 | 8.81 | 3.35 | 4.35 | 4.57 | 3.51 | 3.65 | 3.89 | 3.57 | 3.94 |
| Cytophagia | Bacteroidota | 0.75 | 0.76 | 0.56 | 1.26 | 1.15 | 0.62 | 1.70 | 1.78 | 1.41 | 3.22 | 1.11 | 0.77 | 0.83 | 2.03 |
| Sphingobacteriia |  | 0.83 | 0.81 | 0.64 | 1.97 | 1.70 | 0.73 | 0.95 | 0.72 | 0.64 | 1.28 | 0.48 | 0.42 | 1.06 | 0.90 |
| Chitinophagia |  | 0.22 | 0.26 | 0.22 | 0.63 | 0.39 | 0.26 | 0.44 | 0.61 | 0.61 | 1.05 | 0.41 | 0.21 | 0.34 | 0.68 |
| Other |  | 0.22 | 0.28 | 0.26 | 0.30 | 0.26 | 0.22 | 0.47 | 0.59 | 0.51 | 0.58 | 0.53 | 0.27 | 0.26 | 0.54 |
| Total |  | 2.02 | 2.11 | 1.69 | 4.16 | 3.49 | 1.83 | 3.57 | 3.70 | 3.17 | 6.14 | 2.52 | 1.67 | 2.50 | 4.15 |
| Planctomycetia | Planctomycetota | 1.66 | 1.76 | 2.20 | 1.59 | 1.34 | 1.41 | 2.53 | 1.85 | 1.65 | 1.76 | 1.56 | 1.97 | 1.81 | 1.95 |
| Other |  | 0.10 | 0.08 | 0.08 | 0.07 | 0.09 | 0.06 | 0.43 | 0.47 | 0.46 | 0.43 | 0.29 | 0.07 | 0.08 | 0.45 |
| Total |  | 1.76 | 1.85 | 2.29 | 1.67 | 1.43 | 1.47 | 2.96 | 2.32 | 2.11 | 2.19 | 1.85 | 2.05 | 1.89 | 2.39 |
| unclass. Cyanobacteria | Cyanobacteria | 1.54 | 1.59 | 1.57 | 1.63 | 1.47 | 1.55 | 0.99 | 0.99 | 1.07 | 0.94 | 1.41 | 2.17 | 1.58 | 1.00 |
| Other |  | 0.06 | 0.06 | 0.07 | 0.07 | 0.06 | 0.10 | 0.05 | 0.03 | 0.04 | 0.02 | 0.05 | 0.08 | 0.06 | 0.03 |
| Total |  | 1.60 | 1.65 | 1.65 | 1.70 | 1.53 | 1.64 | 1.03 | 1.02 | 1.11 | 0.96 | 1.45 | 2.25 | 1.65 | 1.03 |
| Blastocatellia | Acidobacteriota | 0.42 | 0.30 | 0.44 | 0.60 | 0.30 | 0.36 | 0.63 | 1.42 | 1.12 | 0.41 | 0.70 | 0.37 | 0.44 | 0.89 |
| Other |  | 0.64 | 0.62 | 0.65 | 0.69 | 0.57 | 0.93 | 0.71 | 0.92 | 0.91 | 0.74 | 0.93 | 0.79 | 0.65 | 0.82 |
| Total |  | 1.06 | 0.92 | 1.09 | 1.29 | 0.87 | 1.29 | 1.34 | 2.33 | 2.03 | 1.14 | 1.64 | 1.16 | 1.09 | 1.71 |
| Pezizomycetes | Ascomycota | 0.20 | 0.29 | 0.05 | 0.43 | 0.48 | 0.14 | 1.70 | 0.74 | 0.23 | 0.76 | 0.24 | 0.24 | 0.24 | 0.86 |
| Other |  | 0.40 | 0.48 | 0.39 | 0.52 | 1.71 | 0.36 | 0.87 | 0.40 | 0.35 | 0.66 | 0.52 | 0.65 | 0.44 | 0.57 |
| Total |  | 0.60 | 0.77 | 0.44 | 0.95 | 2.19 | 0.50 | 2.57 | 1.13 | 0.58 | 1.42 | 0.76 | 0.89 | 0.69 | 1.43 |
| Oligohymenophorea | unclass. Eukaryota | 0.10 | 0.18 | 0.11 | 0.50 | 0.34 | 0.27 | 0.42 | 1.81 | 2.62 | 2.43 | 0.49 | 0.13 | 0.22 | 1.82 |
| unclass. Eukaryota |  | 0.16 | 0.26 | 0.13 | 0.23 | 0.25 | 0.12 | 0.21 | 0.43 | 0.68 | 1.66 | 0.87 | 0.14 | 0.19 | 0.74 |
| Other |  | 0.03 | 0.08 | 0.10 | 0.18 | 0.07 | 0.08 | 0.04 | 0.04 | 0.03 | 0.34 | 0.04 | 0.10 | 0.10 | 0.11 |
| Total |  | 0.29 | 0.53 | 0.34 | 0.90 | 0.66 | 0.46 | 0.67 | 2.28 | 3.32 | 4.43 | 1.40 | 0.37 | 0.51 | 2.67 |
| Gemmatimonadetes | Gemmatimonadota | 0.30 | 0.23 | 0.20 | 0.24 | 0.22 | 0.21 | 1.65 | 1.33 | 1.18 | 1.29 | 1.05 | 0.23 | 0.24 | 1.36 |
| Total |  | 0.30 | 0.23 | 0.20 | 0.24 | 0.22 | 0.21 | 1.65 | 1.33 | 1.18 | 1.29 | 1.05 | 0.23 | 0.24 | 1.36 |
| **REST** | | 4.82 | 5.79 | 4.96 | 5.53 | 5.34 | 5.95 | 5.55 | 6.12 | 6.86 | 6.44 | 6.83 | 6.80 | 5.27 | 6.24 |
| **Classified TPM** | | 41.36 | 37.52 | 39.64 | 36.72 | 37.16 | 33.50 | 41.02 | 39.00 | 38.29 | 36.61 | 36.84 | 30.52 |  |  |

**Table S2**: Taxonomic classification of gene transcripts as a percentage of all classified transcripts (TPM). Only phyla with transcript abundances >1% on the 7-hour averages (dry or watered) are shown. “Classified TPM” percentages indicated in the bottom row are calculated from the total.

## Supplementary figures

| 7 h 1 | 2 h 3 | 3 d 2 | 10 min 3 | 1 d 2 | - | 4 d 3 |
| --- | --- | --- | --- | --- | --- | --- |
| 3 d 3 | 7 d 1 | - | 1 d 1 | 1 h 1 | - | - |
| 1 mo 1 | 7 d 3 | - | - | 2 h 2 | 10 min 1 | 2 d 1 |
| 2 d 2 | - | - | 2 d 3 | 2 mo 2 | 7 h 2 | - |
| 1 d 3 | - | 1 mo 3 | - | 3 h 3 | 2 mo 3 | 1 h 2 |
| 2 h 1 | 3 d 1 | 7 h 3 | 4 d 1 | 4 d 2 | 10 min 2 | 2 mo 1 |
| 1 h 3 | - | - | 3 h 2 | 7 d 2 | 1 mo 2 | 3 h 1 |

**Figure S1**: Spatial distribution of the sampled soils within the sampling plots. The same positions were used for the dry and watered plots. The times for which samples were sequenced are coloured. Refer to Table S1 for more details on the sequenced sectors.


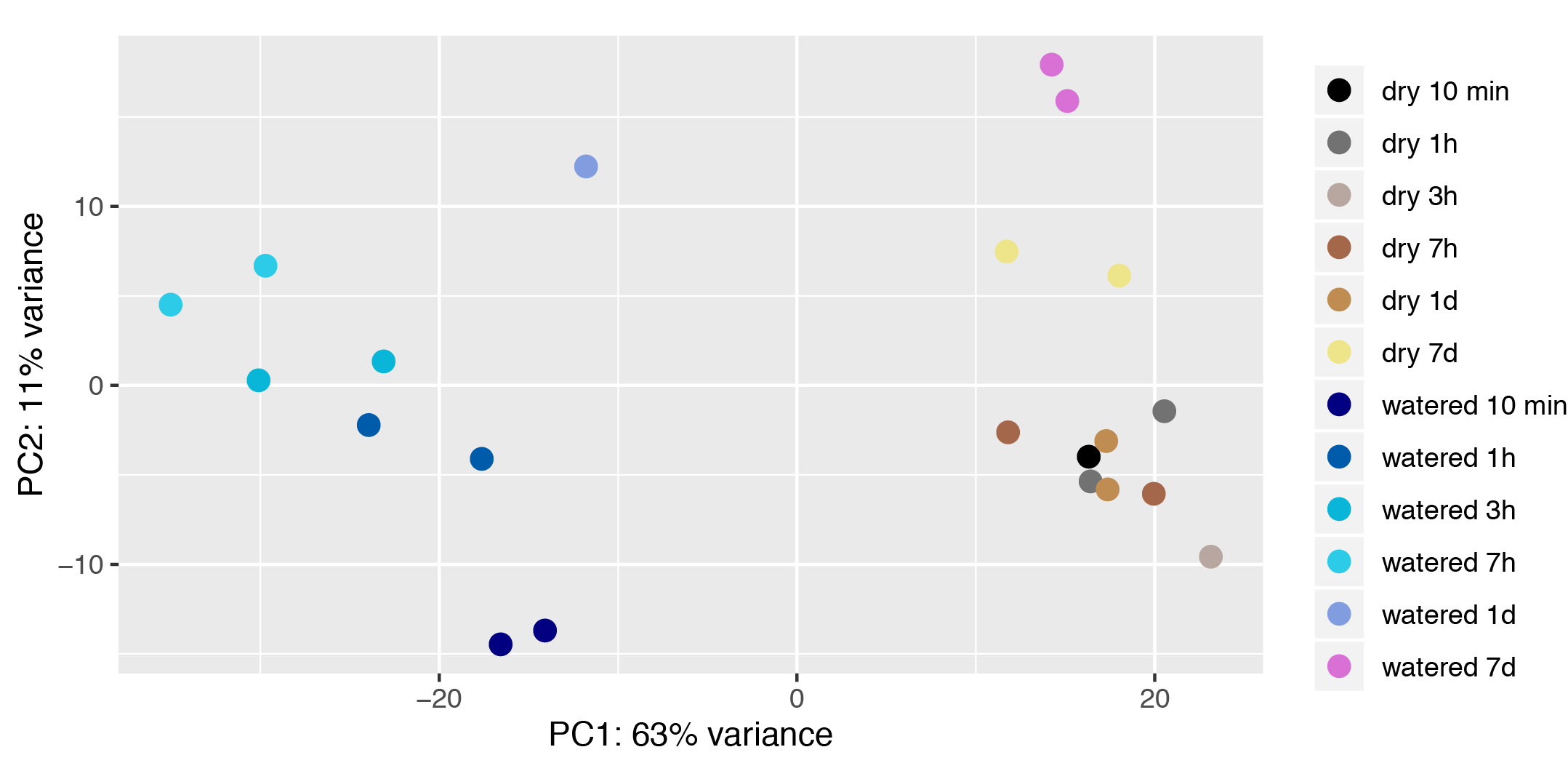


**Figure S2**: principal components analysis of transcriptome reads classified along combined taxonomic (class) and functional (KO) categories. Computed by *DESeq2*.


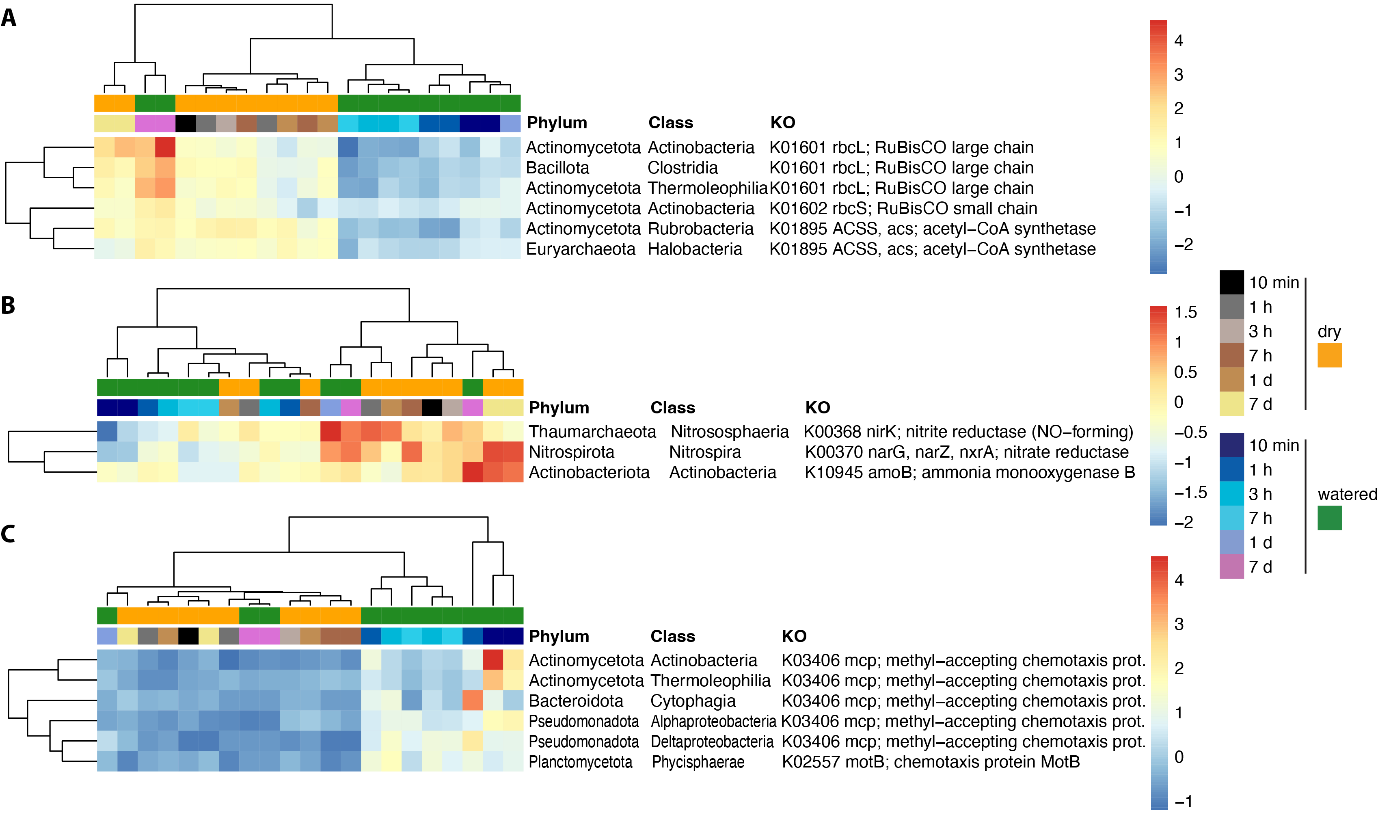


**Figure S3**: A) Carboxylase genes from carbon fixation pathways, B) nitrogen cycling enzyme genes and C) chemotaxis-related genes significantly changing after watering. Transcript data was aggregated along combined taxonomic (class) and functional (KEGG Orthologs) groups for the analysis. Values were normalized using the Variance Stabilizing Transformation (*DESeq* R package). Rows and columns were clustered using *hclust*.


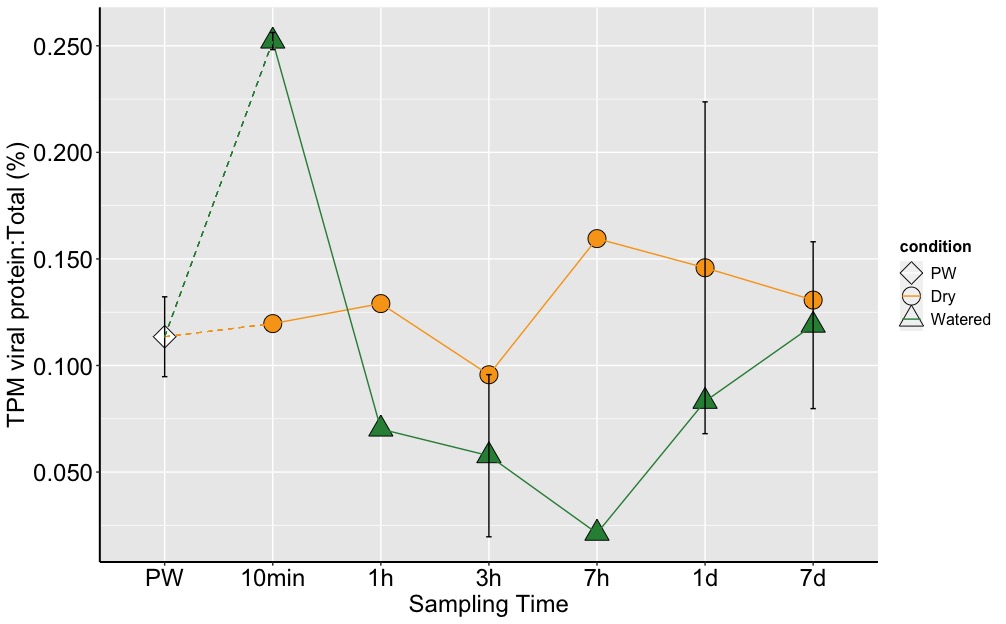


**Supplementary Figure S4.** Temporal changes in viral protein gene transcripts as a fraction of the total (Vp:T ratios; %) for watered and dry (control) soil samples. The PW (Pre-watering) value represents the mean ± sd of Vp:T ratios in all control samples.


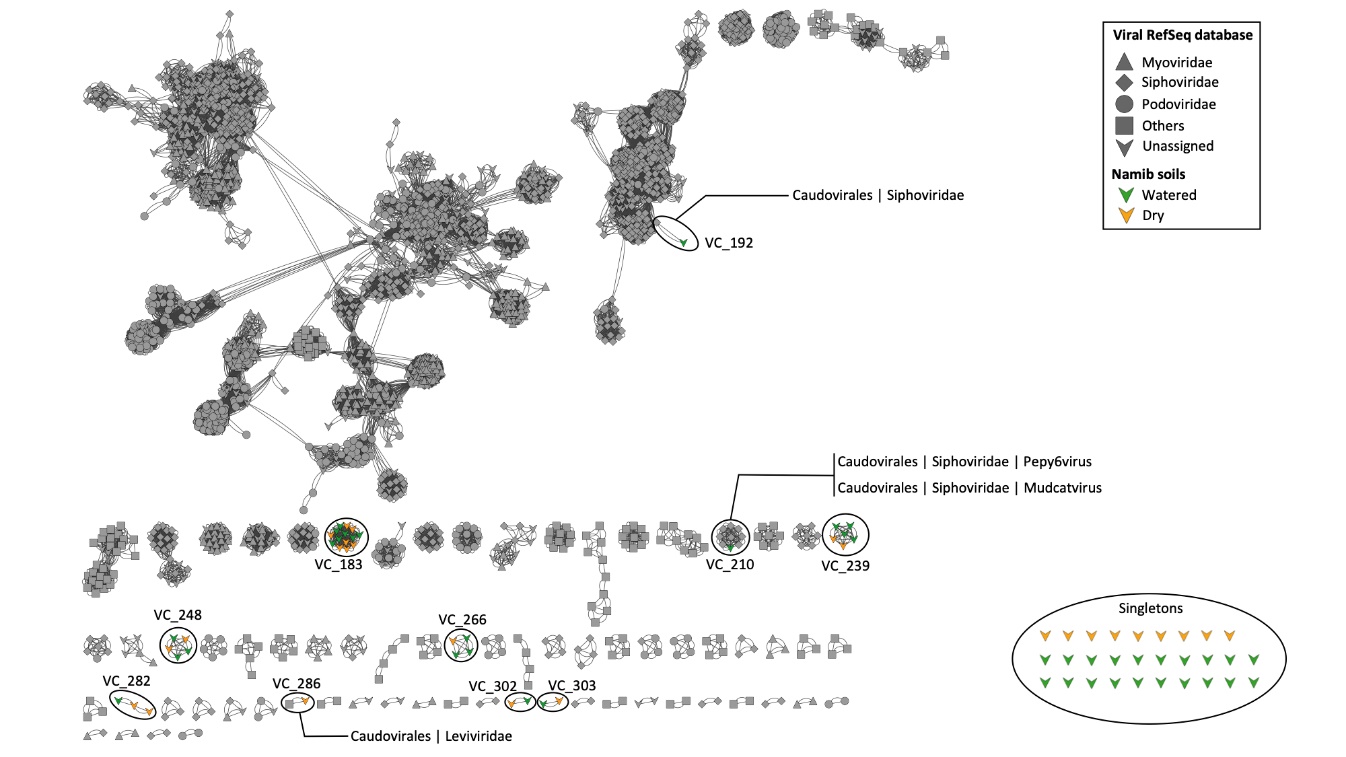


**Supplementary Figure S5.** Network analysis, relating Water Experiment (WE) phage sequences to known viral sequences from the RefSeq Database. Circled clusters present viral contigs identified in this study. Shapes indicate major viral families, RefSeq sequences are in grey and WE contigs are colored (yellow and green for dry and watered samples, respectively). Each node is depicted as a different shape, representing viruses belonging to Myoviridae (triangle), Podoviridae (circle), Siphoviridae (diamond), or uncharacterized viruses (V shape). Single shapes represent viral singletons identified in this study. Edges (lines) between nodes indicate statistically weighted pairwise similarity scores (see Materials and Methods) of ≥ 1.
